# Identifying hotspots of land use cover change under socioeconomic and climate change scenarios in Mexico

**DOI:** 10.1007/s13280-018-1085-0

**Published:** 2018-08-20

**Authors:** Alma Mendoza-Ponce, Rogelio O. Corona-Núñez, Leopoldo Galicia, Florian Kraxner

**Affiliations:** 1 International Institute for Applied Systems Analysis, Schloßpl. 1, Laxenburg, 2361 Vienna, Austria; 2 Procesos y Sistemas de Información en Geomática, S.A. de C.V., Calle 5 Viveros de Petén #18, Col. Viveros del Valle, 54060 Tlalnepantla, Mexico; 3 Departamento de Geografía Física, Instituto de Geografía, Universidad Nacional Autónoma de México, Investigación Científica, Circuito Exterior s/n, Ciudad Universitaria, Coyoacán, 04510 Mexico, Ciudad de México Mexico

**Keywords:** Deforestation, Drivers, Ecosystems, Mexico, Scenarios

## Abstract

This study identifies the hotspots of land use cover change (LUCC) under two socioeconomic and climate change scenarios [business as usual (BAU) and a pessimistic scenario] at the national level for Mexico for three-time periods. Modelling suggests that by 2050 grassland and tropical evergreen forest will be the most endangered ecosystems, having lost 20–33% (BAU) or 43–46% (pessimistic scenario) of their extent in comparison to 1993. Agricultural expansion would be the major driver of LUCC, increasing from 24.4% of the country in 1993 to 30% (BAU) or 34% (pessimistic) in 2050. The most influential variables were distance from roads and human settlements, slope, aridity, and evapotranspiration. The hotspots of LUCC were influenced by environmental constraints and socioeconomic activities more than by climate change. These findings could be used to build proposals to reduce deforestation, including multiple feedbacks among urbanization, industrialization and food consumption.

**Electronic supplementary material:**

The online version of this article (10.1007/s13280-018-1085-0) contains supplementary material, which is available to authorized users.

## Introduction

Land use cover change (LUCC) is the physical expression of human impacts on the landscape. Drivers of LUCC vary in magnitude and location, affecting differentially the processes and patterns of the earth’s surface. An understanding of the socio-ecological drivers of change and how they impact the land systems is necessary to perceive how these changes might affect the system and their tradeoffs (Verburg et al. 2015). The relationship and feedback between LUCC and climate change have attracted attention in recent decades because they are expected to act synergistically to threaten ecosystem services and biodiversity (Beale et al. 2013).

Deforestation processes change over time and space. They are related to underlying causes such as economic, demographic, technological, cultural and political factors (Veldkamp and Lambin 2001; Geist and Lambin 2002). Global drivers of agricultural expansion are related to population growth, changing diets, animal feed and fuel consumption (Foley et al. 2011; Alexander et al. 2015). Therefore, globalization has caused the effects of LUCC processes to differ between countries and within their borders, because of the differences between the location of food and wood production and their consumption (Lambin and Meyfroidt 2011). For example, although tropical regions have become some of the greatest emitters of CO_2_ due to LUCC processes (Houghton et al. 2012), especially from agriculture (Laurance et al. 2014), European countries import large quantities of agricultural products (Porkka et al. 2017).

In Latin America, most agricultural production is destined for domestic markets (Meyfroidt et al. 2013). Therefore, spatially explicit land use models that incorporate the proximate causes of LUCC require an understanding of the agricultural spatial patterns and their dynamics (Veldkamp and Lambin 2001; Verburg et al. 2002). In this context, LUCC models contribute to an understanding of complex socio-ecological systems. These models monitor areas and types of changes which can be incorporated to quantify and qualify the impacts of LUCC on carbon emissions (Houghton et al. 2012), climate (Feddema et al. 2005), ecosystem services and biodiversity conservation (Wu 2013). Also, these models identify spatially the drivers of LUCC and magnitude and intensity of the effects; this information can influence policies for ecosystems management. Consequently, national, regional and local studies are needed to improve the understanding of the LUCC and their effects.

Mexico is one of the countries with the greatest extent of natural vegetation (FAO 2015), and one of the 5 out of the 17 richest countries in terms of biological diversity and endemism (Mittermeier et al. 1997). However, the forest and biodiversity are at risk due to deforestation (e.g. during 2010–2015 the deforestation affected 72 200 ha year^−1^) (FAO 2015). In Mexico there are two principal agricultural management practices: (1) high-technology agriculture linked to industrialized centres and urban areas, and (2) traditional agriculture associated with marginalized communities (López et al. 2001; Currit and Easterling 2009). These constitute a complex framework which makes Mexico an interesting case in understanding the deforestation processes with regard to socioeconomic change and climate change in complex and heterogeneous territories. Therefore, the aims of this study are to contextualize the drivers of change and to determine the hotspots of LUCC under different socioeconomic and climate change scenarios in the short, medium and long term for Mexico. To achieve this goal, two key questions were developed: (1) What are the main drivers of LUCC in Mexico? (2) What ecosystems will be the most threatened by LUCC under diverse socioeconomic conditions and climate change scenarios?

## Materials and methods

### Classes of land uses and covers, and explanatory variables

This study used three national land cover maps (1993, 2002 and 2007) in vector format. The original classification includes more than 70 classes of land uses and covers, but these were aggregated into nine classes: temperate forest, scrubland, hydrophilic vegetation, agriculture, tropical evergreen forest, tropical dry forest, other vegetation such as palms, natural grasslands, and other covers including urban and barren lands.

Selection of socioeconomic, biophysical and climate explanatory variables was based on other deforestation and LUCC studies undertaken at different temporal and spatial scales (Geoghegan et al. 2001; Roy-Chowdhury 2006; Flamenco-Sandoval et al. 2007; Wyman et al. 2008; Currit and Easterling 2009; Ellis et al. 2010; Sahagún-Sánchez et al. 2011; Pérez-Vega et al. 2012) (Table S1). All spatial variables were harmonized under the same projected coordinate system with a Datum WGS 84 and grid cells (1 km × 1 km). The total extent was 1 907 382 km^2^ excluding islands and water bodies.

To assess the likely effect of climate change on LUCC processes, four coupled global atmosphere–ocean general circulation models, GCMs (HadCM3, CGCM2, MK2 and Nies 99), were considered. The selected climate variables were aridity index, potential evapotranspiration and temperature seasonality, all with a spatial resolution of 30 arc sec. Metzger et al. (2013) used these derived climate variables to reconstruct the different ecosystems and ecoregions; this performed better than inclusion of a larger non-processed climate data set, such as the one provided by BIOCLIM, and it explained > 99.9% of the global environmental stratification. The environmental stratification based on these variables has shown high compatibility with other environmental stratifications such as the biomes used to underpin the World Wildlife Fund, ecoregions (Olson et al. 2001), or an updated Köppen map of the world (Peel et al. 2007). Also, these bioclimate indicators were directly related to plant physiological processes and primary productivity (Leathwick et al. 2003).

#### Characterization of temporal and spatial LUCC patterns

The LUCC model was calibrated with the land use and cover maps of the years 1993 and 2002. Transition matrices were built to calculate the rate of change between classes. The LUCC model assesses the contribution of change in area and percentage of the total changes per period. In total, 20 transitions out of 72 were evaluated.

All the predictor variables were categorized to estimate the effect of each one on a specific transition by calculating the probability of absence or presence (Goodacre et al. 1993; Bonham-Carter 1994). The categorization is based on an adaptation from Agterberg and Bonham-Carter’s (1990) method, which consists of creating intervals for every transition, respecting the distribution of the data structure. The resulting ranges are the best fitting curve by straight-line segments that define the curve (Soares-Filho et al. 2009). *Weights of evidence* (WofE) were calculated to evaluate the likelihood of LUCC for each predictor variable (Soares-Filho et al. 2001, 2002). A positive value of WofE indicates that the relationship between a specific transition and the variable is stronger than would normally occur by chance; a negative value indicates that fewer observations occur than random processes. Absolute values from 0 to 0.5 are mildly predictive, from 0.5 to 1 are moderately predictive, from 1 to 2 are highly predictive, and ≥ 2 are extremely predictive (Agterberg and Bonham-Carter 1990; Goodacre et al. 1993; Bonham-Carter 1994).

An absolute weighted mean based on the area of each transition was calculated to compare the importance among variables per transition (Eq. 1). The TWofE expresses the overall effect of each variable on each transition.

1 $$ {\text{TWofE}}_{xy} = \frac{{\mathop \sum \nolimits_{{i_{xy} = 1}}^{{n_{xy} }} |{\text{W}}^{ + }{\text{ofE}}_{{i_{xy} }} | \cdot A_{{i_{xy} }} }}{{{\text{TA}}_{y} }}, $$

where TWofE_*x*,*y*_ is the total W of E_*xy*_ of each variable, *x* is variable, *y* is transition, *A*_*i*_ is area in km^2^ per variable and range and TA_*y*_ is total area per transition (including all the ranges from 1 to *n*).

Correlated variables were excluded from the analysis. The correlation between variables was analysed by Crammer’s index and related to every transition. When the correlation values were high (> 0.5), the variable with the higher WofE was selected for analysis (Soares-Filho et al. 2009).

#### Land use cover change dynamics and scenarios

This paper associates the socioeconomic drivers, the climate elements and the land use change in a single framework. It incorporates the Special Report on Emissions Scenarios (SRES) (IPCC 2000) because they are based on intrinsically linked storylines, socioeconomic projections and climate variables. This contrasts with the shared socioeconomic pathways (SSPs) and the representative concentration pathways (RCPs) which were developed largely independently and may be integrated within several combinations (Kriegler et al. 2012; van Vuuren et al. 2014). This is important because different SSPs and the RCP combinations may build similar scenarios depending on contrasting assumption of land use trends, energy consumption and mitigation policies. In terms of climate, it can be linked to large rates of deforestation due to clearances for crops of biofuels (Popp et al. 2017; Riahi et al. 2017). Consequently, to keep a unifying storyline for this study, the LUCC projections were based on two assumptions regarding socioeconomic and climate change: a business as usual (BAU) scenario based on medium population and economic growth with medium rates of LUCC and B2 climate data (medium rate of change); and a pessimistic scenario based on high population growth and rates of LUCC, and low economic growth, which is in accordance with the A2 climate scenario assumptions (high rate of change). Finally, these scenarios were chosen according to the availability of information regarding bioclimate variables at fine spatial resolution (Metzger et al. 2013).

Projection of the assumptions of LUCC used a Markov change matrix and its modification. The BAU scenario used the rates of change and LUCC trajectories recorded for the period 1993–2002. However, for the pessimistic scenario, the magnitudes of the trajectories were adjusted particularly for the transitions to agriculture and other covers (urban). These modifications were based on the storylines and assumptions of the pessimistic scenario which includes a high population growth and slow growth of gross domestic product (GDP). These changes were incorporated in a lineal relationship to project their effects on areas of agricultural and urban lands. The LUCC model was then updated with the socioeconomic and climate variables for each scenario to simulate future land covers (2020, 2050 and 2080). Climate information was specific to each model and scenario (Table S1). The model was repeated using four GCMs (HadCM3, CGCM2, MK2 and Nies 99) for each time slice and scenario. More recent scenarios were not used, since the dates and models used in this study reflect current trends in population growth, environmental policies and socioeconomic conditions.

### Model validation and uncertainty estimations

The trained model was projected to the year 2007. The simulated map was validated to reflect the reliability of the model. A perfect simulation occurs when every grid cell is identical to the observed map (Pontius et al. 2001). The model was evaluated in terms of accuracy in location and in quantity of change between the observed and modelled maps for the year 2007. Model validation used two methods: *reciprocal similarity* (Soares-Filho et al. 2009), a modification of the Kappa Fuzzy (κ*Fuzzy*) proposed by Hagen (2003), taking into account the fuzziness of location and category within a cell neighbourhood over different resolutions; and the *figure of merit*, used to detect the differences and similarities between the evaluated maps and expressed as the percentage of the intersection of the observed and simulated changes of every cover in relation to its own area (Perica and Foufoula-Georgiou 1996). If the model prediction is perfect, the figure of merit is 100%. On the contrary, if the prediction fails completely it is zero (Pontius et al. 2008). Based on the figures of merit, Pontius and Millones (2011) proposed the concepts of agreement and disagreement in allocation and quantity between the observed and modelled maps; for this, quantity of disagreement is defined as the amount of difference between the observed map and a simulated map that is due to the less-than-perfect match in the proportions of the categories. Allocation disagreement is defined as the amount of difference between the observed and the simulated maps in the spatial allocation of the categories, given the proportions of the categories in the two maps. For more details about these indexes, refer to Pontius and Millones (2011).

The uncertainty of the resulting maps was evaluated by quantifying the agreement between the four maps (one for each GCM) for each scenario and time frame. This agreement ranked from 0 to 100, wherein a value of 100 was for cells in which the four GCMs projected the same transition or permanence, a value of 75 where three out of four models coincided and 50 where only two models showed an agreement in the modelled transitions. Consequently, it was possible to assess the performance of the model with the different GCMs for the LUCC trajectories.

## Results

### Past and future LUCC trajectories

Agricultural expansion was the principal cause for ecosystem change. Its expansion explained ~ 49% and ~ 65% of the conversion of ecosystems for 1993–2002 and 2002–2007, respectively; agricultural cover showed a constant expansion at 28 000 km^2^ year^−1^ during 1993–2002 and 16 000 km^2^ year^−1^ during 2002–2007 (Tables 1, 2). Agricultural expansion was mainly on the east coast and the south-eastern part of the country (particularly in the State of Chiapas) and along the Trans-Mexican Volcanic Belt where it was related to highly populated areas.

**Table 1:** Transition matrices during the period 1993–2002 (km^2^). *TF* temperate forests, *S* scrublands, *HV* hydrophilic vegetation, *A* agriculture, *TEF* tropical evergreen forests, *TDF* tropical dry forests, *G* grasslands, *OV* other vegetation, *OC* other covers

1993	2002										
	TF	S	HV	A	TEF	TDF	G	OV	OC	Total 1993	Loss
TF	320 305	1209	7601	16 230	1035	4758	2376	16	79	353 609	33 304
S	1479	538 949	318	14 148	0	3185	4172	1151	498	563 900	24 951
HV	137	294	8382	826	100	77	85	80	109	10 090	1708
A	10 896	6599	786	424 220	4893	12 367	2537	431	1855	464 584	40 364
TEF	575	0	302	10 447	92 988	449	108	4	119	104 992	12 004
TDF	6130	2440	112	19 245	1532	201 765	430	40	235	231 929	30 164
G	3112	3344	81	5146	121	296	115 886	181	118	128 285	12 399
OV	97	949	240	1467	1	130	728	27 249	267	31 128	3879
OC	44	243	83	1121	18	80	92	180	17 004	18 865	1861
Total 2002	342 775	554 027	17 905	492 850	100 688	223 107	126 414	29 332	20 284	1 907 382	
Gain	22 470	15 078	9523	68 630	7770	21 342	10 528	2083	3280		
Net balance	− 10 834	− 9873	7815	28 266	− 4304	− 8822	− 1871	− 1796	1419

**Table 2:** Transition matrices during the period 2002–2007 (km^2^). *TF* temperate forests, *S* scrublands, *HV* hydrophilic vegetation, *A* agriculture, *TEF* tropical evergreen forests, *TDF* tropical dry forests, *G* grasslands, *OV* other vegetation, *OC* other covers

2002	2007										
	TF	S	HV	A	TEF	TDF	G	OV	OC	Total 2002	Loss
TF	325 652	779	237	11 112	346	3503	994	65	87	342 775	17 123
S	853	538 807	188	10 531	0	284	1767	833	764	554 027	15 220
HV	11	124	16 476	846	238	74	53	46	37	17 905	1429
A	9685	5561	770	455 809	4474	10 651	2453	437	3010	492 850	37 041
TEF	335	0	243	6760	92 515	535	106	4	190	100 688	8173
TDF	3208	468	166	15 886	883	201 912	202	15	367	223 107	21 195
G	1533	3633	130	5793	137	236	114 617	105	230	126 414	11 797
OV	5	497	76	1243	6	40	262	26 978	225	29 332	2354
OC	17	151	60	845	34	55	110	92	18 920	20 284	1364
Total 2007	341 299	550 020	18 346	508 825	98 633	217 290	120 564	28 575	23 830	1 907 382	
Gain	15 647	11 213	1870	53 016	6118	15 378	5947	1597	4910		
Net balance	− 1476	− 4007	441	15 975	− 2055	− 5817	− 5850	− 757	3546		

**Fig. 1:**
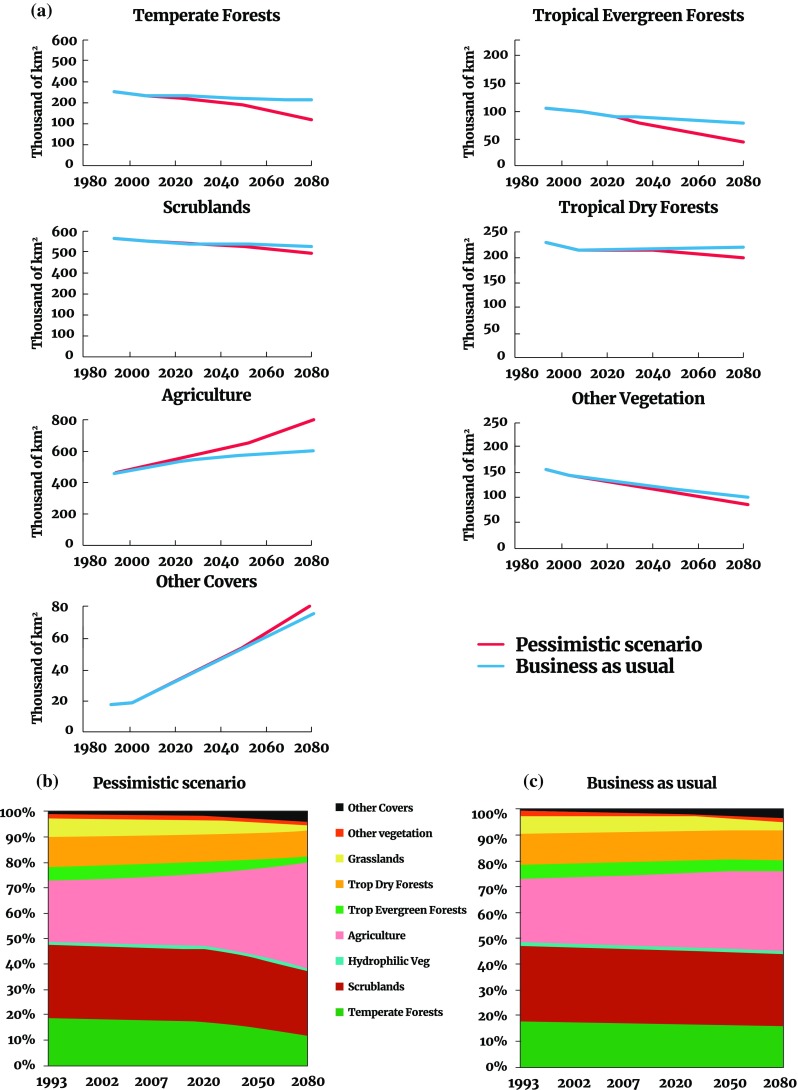
**a** Past and future trends of the principal LUCCs in Mexico under CC scenarios by land use and land cover, **b** percentage of surface of each land use and land cover in the past and the future under a pessimistic scenario and **c** percentage of surface of each land use and land cover in the past and the future under the business as usual (BAU) scenario

During the period 1993–2002, loss of area was greatest in temperate forest (1204 km^2^ year^−1^), scrubland (1097 km^2^ year^−1^) and tropical dry forest (980 km^2^ year^−1^). As a function of the area in 1993, temperate forest by 2002 had lost 3.1%, tropical dry forest 3.8% and tropical evergreen forest 4.1% (Table 1). During 2002–2007, loss of area was greatest in natural grassland (1170 km^2^ year^−1^) and tropical dry forest (1163 km^2^ year^−1^) and this was also the largest proportional loss (4.6% of grassland and 2.6% of tropical dry forest) in relation to their extent in 1993 (Fig. 1).

#### Socioeconomic variables

Deforestation was principally related to socioeconomic variables. In temperate forest and scrubland, the most important socioeconomic variables related to the LUCC processes were distance from human settlements and roads followed by population density, GDP and marginalization (Table 3). In contrast, transition to agricultural land from grassland was more closely associated with biophysical variables (Table 3). Regarding the relative WofE by ranges, distance from human settlements (< 2 km) was strongly correlated with changes to agricultural area. This relationship was found in temperate forest, scrubland, tropical dry forest and grassland (WofE > 1.0) (Table S2). Proximity to roads (< 1 km) was an important driver for agricultural activities in all the natural covers (WofE ≥ 0.79) (Table S1). Population density (< 200 inhabitants km^−2^) was linked to conversion from temperate forest and tropical dry forest (WofE = 0.86 and 1.8) to agricultural land, whereas at higher population densities (≥ 500 inhabitants km^−2^) the strong link was to conversion from tropical evergreen forest and scrubland (WofE = 1.9 and 2.5) (Table S2). The National Index of Marginalization was also a significant factor in agricultural and urban expansion. Municipalities with medium and high marginalization were associated with agricultural expansion in temperate forest, tropical evergreen forest and tropical dry forest (WofE ≥ 0.60, Table S2), whereas municipalities with very low or low marginalization favoured urban sprawl (WofE = 0.90 and 1.44). GDP and GDP per capita were similarly influential. For example, poor municipalities (GDP 400–2500 million Mexican pesos) undertook more transitions to agricultural land, whereas richer municipalities (> 5100 million Mexican pesos) were related to the expansion of urban cover. Protected areas were effective in restricting change to agricultural activities in tropical evergreen forest (WofE = 1.86). Proximity to rivers had little influence on expansion of agricultural activities (WofE ≤ 0.41, Table S2).

**Table 3:** TWofE values of socioeconomic and biophysical forces. *TF* temperate forests, *A* agriculture, *S* scrublands, *TEF* tropical evergreen forests, *TDF* tropical dry forests, *G* grasslands

	TF to A		S to A		TEF to A		TDF to A		G to A	
	1993–2002	2002–2007	1993–2002	2002–2007	1993–2002	2002–2007	1993–2002	2002–2007	1993–2002	2002–2007
Socioeconomic										
Index of marginalization	0.19	0.18	0.27	0.29	0.19	0.21	0.18	0.04	0.44	0.39
Distance to human settlements	0.61	0.51	0.69	0.73	0.29	0.34	0.35	0.41	0.42	0.55
Distance to roads	0.47	0.48	0.59	0.36	0.47	0.45	0.32	0.38	0.64	0.60
Distance to NPAs	0.23	0.24	0.63	0.43	0.06	0.11	0.24	0.09	0.80	0.64
GDP^a^	0.54	0.84	0.38	0.51	0.64	0.71	0.46	0.29	1.12	0.65
Population^a^	0.18	0.18	0.12	0.13	0.82	0.11	0.13	0.12	0.14	0.27
Biophysical										
Altitude	0.23	0.28	0.54	0.37	0.13	0.30	0.27	0.14	0.98	0.62
Slope	0.46	0.59	0.55	0.43	0.16	0.13	0.36	0.36	0.49	0.52
AI	0.29	0.24	0.72	0.52	0.37	0.28	0.29	0.15	0.94	0.62
PET	0.46	0.53	0.29	0.28	0.34	0.23	0.34	0.32	1.06	0.63
TSD	0.47	0.27	0.30	0.43	0.41	0.29	0.42	0.28	1.09	0.57

^a^For GDP, per capita GDP, population or population density variables only was selected one among these combination depending on their WofE and the correlation before modelling. The selection varied according to every transition

#### Biophysical variables

Topographical features, such as slope and altitude, were the main factors affecting the location of the deforestation. Agricultural expansion occurred mainly on gentle slopes and at the lower limit of the natural altitudinal distribution. For example, deforestation of temperate forest was strongly associated with slopes ≤ 2° (WofE = 2.2) and with altitudes < 500 m a.s.l. (WofE = 3.6, Table S2). Climate was also important. For example, sites with low potential evapotranspiration (< 1000) were strongly related to change from temperate forest (WofE = 1.9) and grassland (WofE = 1.3) to agricultural land (Table S2), whereas this association was less evident in tropical dry and evergreen forest. Areas with less aridity (more water availability) were more prone to change to agricultural cover, especially from scrubland and grassland (Table S2).

### LUCC dynamics and future scenarios

Agricultural activities and other covers consistently increased in area from the 24.4% of the Mexican territory recorded in 1993 to 30.5% by 2050 with the BAU or 34.1% with the pessimistic scenario, and by 2080 to 31.3% with the BAU or 41.7% with the pessimistic scenario (Fig. 1). Other covers such as cities will increase from 1% of the national territory in 1993 to 2.9% by 2050, to 4.0% for BAU and 4.3% for the pessimistic scenarios by 2080.

**Fig. 2:**
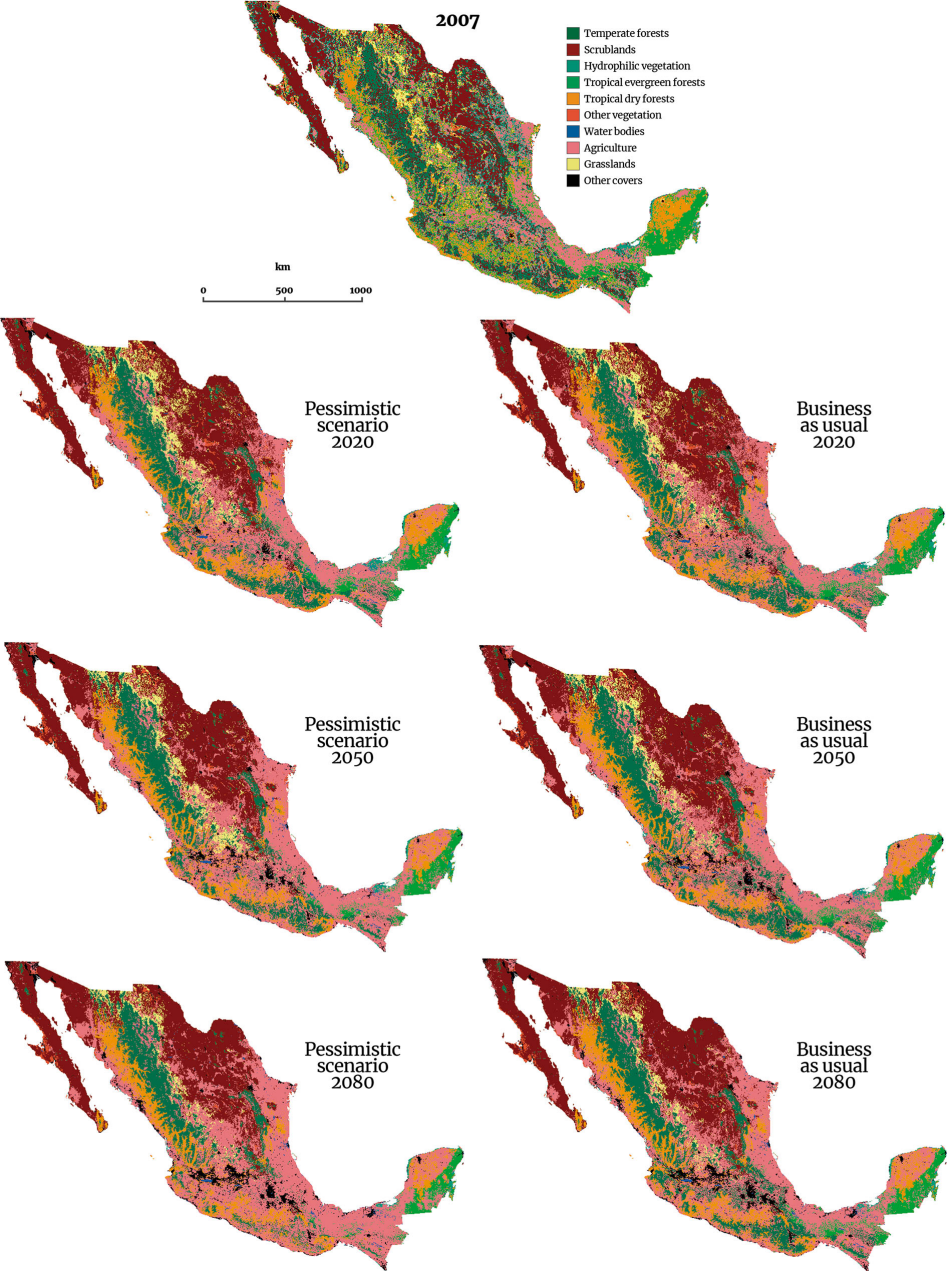
LUCC maps in 2007 and 2020, 2050 and 2080 under pessimistic or BAU scenarios (GCM). Growth of agricultural land occurs along the coast of the Gulf of Mexico, the northern central region and the State of Chiapas, where temperate forests, tropical evergreen forests and natural grasslands are mainly distributed

All the natural covers decreased significantly between 1993 and 2002: temperate forest by 1204 km^2^ year^−1^, scrubland by 1097 km^2^ year^−1^, and grassland by 980 km^2^ year^−1^. Between 2002 and 2007, natural grassland showed the highest rate of loss, at 1170 km^2^ year^−1^. By 2050, the area of grassland would be only 43% of its area in 1993, and tropical evergreen forest would cover 4.7% (BAU) or 3.9% (pessimistic scenario) of the land, suggesting a reduction of between 20 and 33%. By 2080, under the pessimistic scenario, tropical evergreen forest may account for only 2.5% of Mexico, i.e. 45% less than its extent in 1993 (Fig. 1). By 2050, agricultural land would increase from 24.4% in 1993 to 30% (BAU) or 34% (pessimistic scenario), suggesting that by 2080 31–42% of Mexico could be dominated by anthropogenic covers (Fig. 2).

### LUCC model validation and uncertainty in LUCC projections

**Fig. 3:**
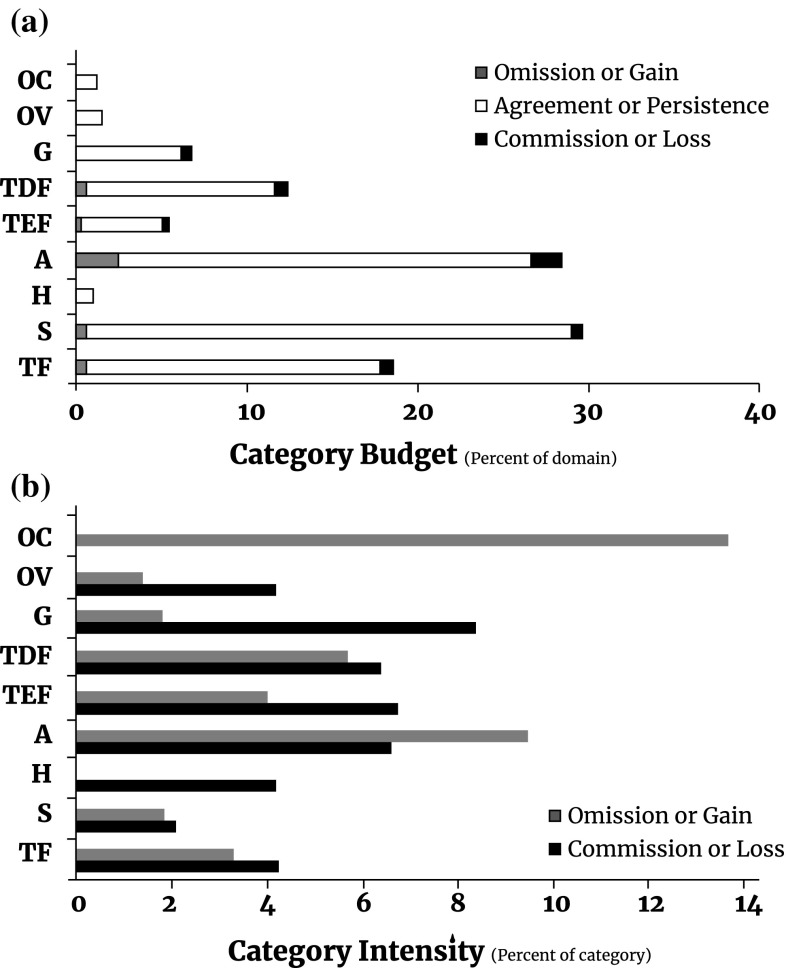
Figures of merit show quantity and allocation percentage of correct and error of the LUCC model according to the observed map vs simulated map. **a** Agreement versus disagreement, **b** the category intensity refers to the percentage of omission or commission in each category. *TF* temperate forests, *S* scrublands, *HV* hydrophilic vegetation, *A* agriculture, *TEF* tropical evergreen forests, *TDF* tropical dry forests, *G* grasslands, *OV* other vegetation, *OC* other covers

The similarity index suggests that the simulated and observed maps reached 70% of the similarity within a window of three cells and 90% within nine cells. According to the figures of merit (Pontius et al. 2008; Pontius and Millones 2011), the *κ* value is 94%, with disagreement values of allocation and quantity of 4% and 1%, respectively. Agricultural cover shows the highest disagreement, while other covers show the highest omission error (Fig. 3). Scrubland and tropical dry forest were the natural covers that showed the highest performance in modelling.

**Fig. 4:**
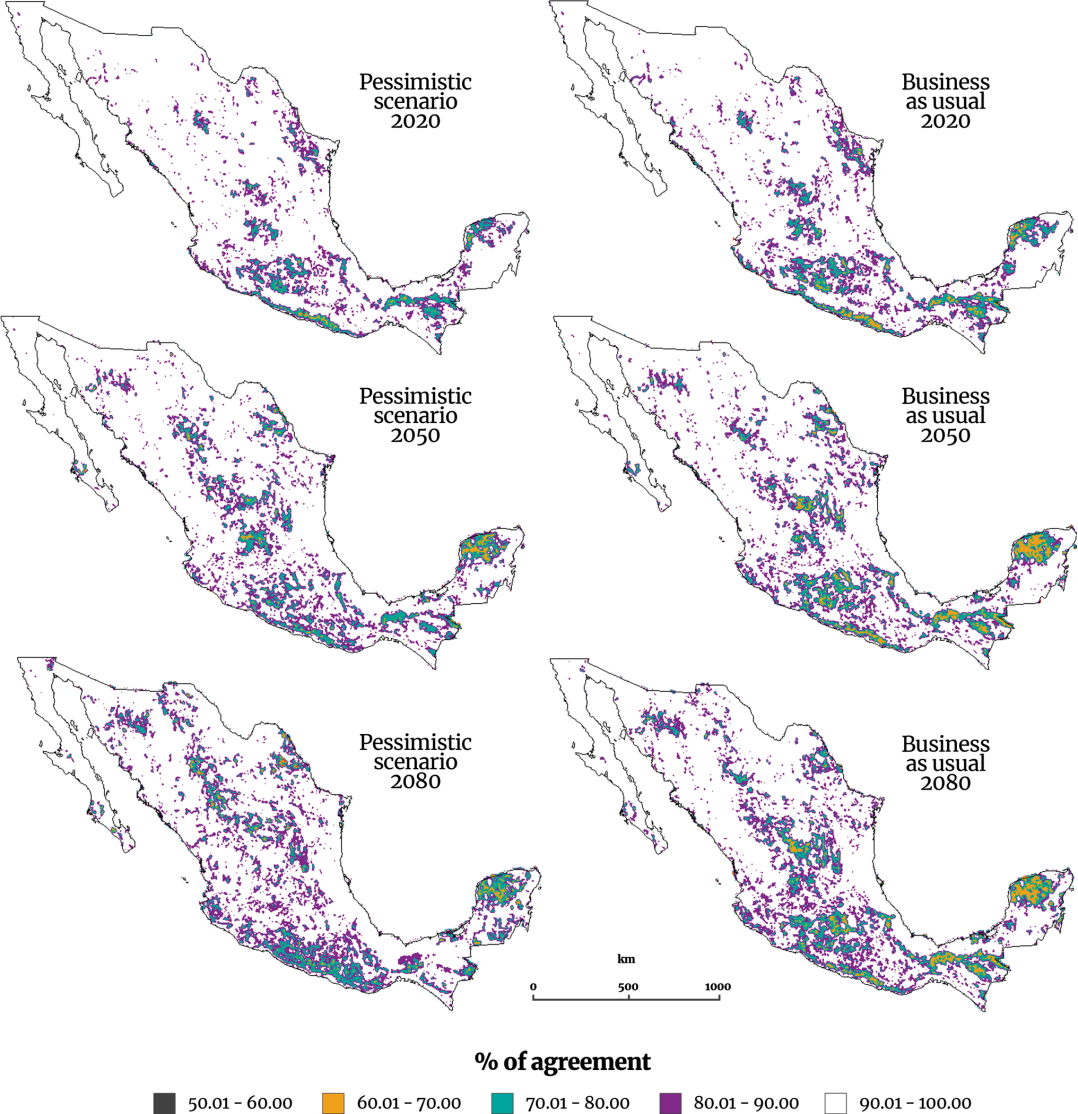
Agreement in projected changes from natural covers to anthropogenic covers between four GCMs. Grey areas showed 100% agreement in projecting deforestation; orange, purple and blue areas show 90–60% agreement

The model suggests differences in the LUCC projections across the Mexican territory. The LUCC models generally agreed among the different GCMs in the northwest of the country and the northern lowlands. However, there was greater disagreement among the GCMs with regard to the Yucatan Peninsula, in the south of the country, and to the Southern Pacific Coast in the states of Michoacán, Guerrero, Oaxaca, and Chiapas, especially by 2080 (Fig. 4). For 2020, there was 100% agreement in 82% (BAU) and 86% (pessimistic scenario) of the total area, but this agreement had decreased by 2050 to 78% (BAU) and 80% (pessimistic scenario), and by 2080 to 73% and 74%.

## Discussion

### LUCC magnitude and trajectories

LUCC and climate change are major drivers of global environmental change. They modify the distribution and fragmentation of the natural vegetation and thereby affect environmental services and biodiversity. Annual rates of deforestation in Mexico dropped by 50% during 2010–2015, in contrast to increases in some other countries, particularly those associated with the expansion of soy crops and pasture for cattle (Gollnow and Lakes 2014; Harfuch et al. 2016). Although FAO (2015) reported deforestation rates for Mexico of 1904 km^2^ year^−1^ in 1990–2000 and 1358 km^2^ year^−1^ in 2000–2010, the present study suggests higher rates, e.g. 2662 km^2^ year^−1^ for 1993–2002 and 1870 km^2^ year^−1^ for 2002–2007. This results from the inclusion of natural vegetation covers that are generally excluded from the analysis because they do not follow the FAO definition of forests (2010). The reduction of forest loss in Mexico shows a pattern similar to the forest recovery in other regions in Latin America, where forest recovery may be favoured by socioeconomic factors such as international remittances, migration, urban/rural population change, rural abandonment and accompanying urbanization and industrialization (Aide and Grau 2004; Grau and Aide 2008; Bonilla-Moheno et al. 2012).

Deforestation rates can hide important losses, particularly those related to the heterogeneity of the LUCC process. For example, during the period 1993–2002, temperate forests and scrublands showed the largest losses. Grasslands and tropical evergreen forests showed the highest proportional loss in relation to their extent in 2007. Since scrubland and grasslands are not considered as forest (FAO 2010), their losses are sometimes overlooked. However, scrubland is the most widespread natural cover in Mexico (Rzedowski 2006; Alanís-Rodríguez et al. 2015) and is undergoing one of the largest rates of depletion in Mexico (Velázquez et al. 2003).

### LUCC drivers

Agricultural expansion affects the natural ecosystems in Mexico (Palacio-Prieto et al. 2000). Diversity in deforestation patterns across the country is related to cultural and socioeconomic activities that differ among ecosystems (Burgos and Maass 2004). In Mexico the spread of agriculture is mainly for subsistence (SAGARPA and FAO 2012), whereas in the Amazon region and Southeast Asia commercial agriculture for international markets is the main driver of deforestation (Hosonuma et al. 2012). Over the past 30 years, industrialized society on the global scale has been experiencing a new model of economic growth whose core aim was to foster a culture of freedom based on technological innovations, resource extraction and entrepreneurship (i.e. open ecology sources, intensive agriculture) (Petropulou 2016). According to Castells et al. (2012), industrialized society has somehow favoured the waves of deregulation, privatization and liberalization, which have been the main objectives of the neo-liberal agenda since the 1980s, and which have disproportionately affected poorer and more marginalized people (Petropulou 2016). Therefore, socioeconomic forces such as population density (Mas et al. 2009), incomes (Vaca et al. 2012; Corona et al. 2016), marginalization and distance from currently existing land uses and covers can be important forces of local and regional LUCC (Sahagún-Sánchez et al. 2011; Kolb et al. 2013). The results of the present study suggest that agricultural expansion is driven by medium to high marginalization, as has also been found in San Luis Potosí, central Mexico (Sahagún-Sánchez et al. 2011) and southern states such as Oaxaca, Veracruz and Chiapas (Bonilla-Moheno et al. 2012; Corona et al. 2016). Rates of transition from forest to agriculture can be high in areas with medium population density. According to Corona et al. (2016), agricultural expansion, mainly for subsistence, is observed in poor municipalities with rural communities, and these tend to have low and medium population densities.

In contrast, high population densities are related to urban expansion, in the present study and elsewhere (Svirejeva-Hopkins and Schellnhuber 2008; Seto et al. 2012). This rapid increase in urban population is mainly due to large-scale migration of people from rural areas and smaller towns to bigger cities in search of better employment opportunities and better quality of life. Urban sprawl has resulted in loss of productive agricultural lands, open green spaces and surface water bodies (Castells et al. 2012). Growing populations are likely to exert pressure to clear forests, primarily because urbanization raises consumption levels and increases the demand for agricultural products (Satterthwaite et al. 2010). Urban consumers generally eat more processed foods and animal products than do rural consumers, thereby causing an increase in the commercial production of crops and livestock supported by the national or international supply chain. This relationship is expected to increase in the near future, considering the current population growth and the changes in food consumption in Mexico (Ibarrola-Rivas and Granados-Ramírez 2017). Across Latin America, the urban population is expected to grow (Inostroza et al. 2013), and this will increase pressure on tropical forests as has already occurred in Africa and Asia (Seto et al. 2012). As a result, if there are no improvements in the yields from Mexican agriculture, agricultural expansion would try to fulfil the demand for resources associated with urban population growth. Because much of the agriculture is of subsistence, Mexico should implement sustainable techniques of production to increase the yields. Otherwise, Mexico would depend on higher imports, impacting on food security. For instance, the OECD–FAO (2017) suggests that Mexico will keep being dependent on maize, dairy products and oilseed imports.

In identifying the causes of deforestation and the influence of climate change and socioeconomic factors, it is necessary to prioritize the hotspots of change. LUCC processes differ across Mexico. For example, the reduction in deforestation in northern areas (Chihuahua and Coahuila) noted here and by Bonilla-Moheno et al. (2012) may be because the North American Free Trade Agreement between Mexico, the USA and Canada has encouraged the inhabitants to engage in the textile industry rather than in agriculture (Currit and Easterling 2009). On the other hand, in municipalities with higher levels of GDP a reduction in agricultural expansion was accompanied by expansion of human settlements and urban areas. Poorer states such as Guerrero, Oaxaca and Chiapas are expected to increase their population, but without any associated economic growth. This will increase pressure on the ecosystems, especially in tropical dry forests (Corona et al. 2016) and tropical evergreen forests linked with past and probable future deforestation rates. Therefore, to identify the most profitable agricultural practices to decrease deforestation and allow forest recovery it is necessary to build proposals that include multiple feedbacks among urbanization, industrialization, market-oriented agricultural production and industry-based agro-technology (García-Barrios et al. 2009).

In Mexico, human settlements are embedded at all scales in forested areas (García-Barrios et al. 2009). Access to forested areas along roads is among the most significant factors contributing to deforestation across the tropics. Regions with high accessibility to forests and high population densities have reduced areas of primary forest, often limited to mountainous regions (Porter-Bolland et al. 2007; Corona et al. 2016). In all ecosystems, proximity to roads, rivers and human settlements favours change to agricultural activities. Social and economic driving forces include the low profitability and productivity of farming, and new transport infrastructure and especially roads, which have rapidly altered many rural landscapes (Caraveli 2000; Petropulou 2016). Consequently, the creation of roads or their improvement is associated with forest loss, reduction of transport costs and increased access to markets. Therefore, further studies should look into the role of the socioeconomic drivers to understand the spatio-temporal dynamics of the LUCC. Answering questions such as: Do the road expansion and GDP are causes or consequences of agricultural and urban sprawl? In contrast, conservation policies such as the presence of protected areas hinder change to agricultural cover, especially in tropical evergreen forest (Figueroa and Sánchez-Cordero 2008). However, national protected areas have not been sufficient to preserve the remnants of the ecosystems, which have been significantly and continuously reduced (Flamenco-Sandoval et al. 2007). Also, conservation policies must take into account other ecosystems such as tropical dry forest or scrubland, which are under-represented in the natural protected areas (Koleff et al. 2009).

### LUCC and climate

Diverse biophysical variables can influence LUCC processes in Mexico over various spatial scales (Kolb et al. 2013). The present study supports the conclusion from a study performed at local scale (Corona et al. 2016) that lower altitudes and gentle slopes favour transition to agricultural and other covers such as cities. Other biophysical variables, such as the aridity index and the potential evapotranspiration influence the extent to which agricultural land is established and expanded (Zomer et al. 2014). For example, water availability (low potential evapotranspiration and high aridity index) was the main factor correlated to agricultural expansion. This explains why most of the deforestation was observed in the dry sub-humid and humid areas, which in turn can be related with the relationship between low production yields and water stress (Bannayan et al. 2010). Therefore, national studies should investigate the connections among climate variables, management and production yields to implement appropriate strategies of mitigation and adaptation under climate change conditions (Pittelkow et al. 2014) with particular focus on temperate and arid and semiarid ecosystems (Leemans and Eickhout 2004).

The pessimistic scenario poses the greater challenge not only because of the new climate conditions but also due to the increasing demands of a growing population. Scrubland will expand to the detriment of temperate forest and natural grassland, as has happened in California (Shaw et al. 2011). This might reinforce the LUCC processes in suitable (more humid) ecosystems, particularly to establish agricultural practices. Therefore, water availability will be the major driver of Mexican agriculture, which in turn would influence the LUCC processes. Further studies should focus on yields and their relationship to LUCC and biophysical variables. This will help to improve agricultural management in specific areas such as the semiarid region (Herrera-Pantoja and Hiscock 2015).

## Conclusions

This study is the first at national level in Mexico that integrates the major drivers of environmental change to quantify the historical and future impacts of LUCC under socioeconomic and climate change scenarios. The result of this work provides spatial information to identify the hotspots of LUCCs. It can guide strategies for biodiversity or ecosystem services conservation through spatial prioritization. Temperate forest, natural grassland and tropical evergreen forest will be the land covers most affected by LUCC. Moreover, tropical dry forest and natural grassland will also be endangered as a result of lack of adequate policies for their conservation because these natural covers are under-represented in the national protected areas. Socioeconomic elements, such as proximity to human settlements or roads, and biophysical variables such as altitude, slope and potential evapotranspiration influence agricultural expansion. Further studies at regional or local scales should incorporate spatial information about migration from rural areas to cities, which could lead to the abandonment of agricultural land and hence to the regeneration of ecosystems.

## Electronic supplementary material

Below is the link to the electronic supplementary material.

Supplementary material 1 (PDF 1187 kb)
