# Supplementary material for: Identifying hotspots of land use cover change under socioeconomic and climate change scenarios in Mexico
Source: Ambio. 2018 Aug 20;48(4):336–49. doi: 10.1007/s13280-018-1085-0 (PMC6411666; doi:10.1007/s13280-018-1085-0)
Supplement: Supplementary file 1 — Supplementary material 1 (PDF 1187 kb) [file 13280_2018_1085_MOESM1_ESM.pdf]

Ambio

Electronic Supplementary Material

This supplementary material has not been peer reviewed.

Title: Identifying Hotspots of Land Use Cover Change Under Socioeconomic and Climate Change Scenarios in Mexico

Table S1. Inputs of the LUCC model.

Biophysical				Socioeconomic			
Map	Original scale or resolution	Year	Source	Map	Scale-resolution	Year	Source
Land use and land cover	1:250,000	1993<br>2002<br>2007	INEGI (2001)<br>INEGI (2005)<br>INEGI (2008)	Roads (Highways)	1:1,000,000	2008	Digital Chart of the World (1985)
Altitude and slope	1:100,000	-	INEGI (2000)	Natural Protected Areas	1:400,000	2012	CONANP (2012)
Soil classification	1:250,000	-	INEGI (2002)	Human settlements	1:50,000	2005	INEGI (2008)
Aridity Index<br>Potential Evapotranspiration<br>Temperature Seasonality	30 arc sec	Current<br>2020<br>2050<br>2080	Metzger et al. (2013)	Index of marginalization**	Municipality	1995<br>2000<br>2005<br>2010	CONAPO (1995)<br>CONAPO (2000)<br>CONAPO (2005)<br>CONAPO (2010)
				Population and population density	Municipality	1993<br>2002<br>2007	INEGI, CONAPO, COLMEX (2006)
					Municipality	2020<br>2050<br>2080	Mendoza-Ponce (2016)
Rivers	1:400,000	Current	Maderey and Torres-Rouata (1990)	Gross Domestic Product	Municipality	1993<br>2002<br>2007	Mendoza-Ponce (2016)
						2020<br>2050<br>2080	

**Index of marginalization is a national index, which includes socioeconomic information related to income, health, housing and education. For more information about how the marginalization index was calculated see CONAPO (2011).

References

CONAPO, 1995. Índice de Marginación Municipal [online]. Available from http://www.conapo.gob.mx/es/CONAPO/Indices_de_marginacion_1995. [Accesed 18 June 2013].

CONAPO, 2000. Índice de Marginación [online]. Available from http://www.conapo.gob.mx/es/CONAPO/Indices_de_marginacion_2000. [Accesed 12 June 2013].

CONAPO, 2005. Índice de Marginación. Available from http://www.conapo.gob.mx/es/CONAPO/Indices_de_marginacion_2000_. [Accesed 12 June 2013].

CONAPO, 2010. Indice de Marginación Municipal. Available from http://www.conapo.gob.mx/es/CONAPO/Indices_de_Marginacion_2010_por_entidad_federativa_y_municipio. [Accesed 12 June 2013], Mexico.

CONAPO, 2011. Index of marginalization for Mexican states and municipalities. Consejo Nacional de Población (CONAPO), Mexico, p. 54 (in Spanish).

Digital_Chart_of_the_world, 1985. Red de carreteras (1: 1000000). México.

INEGI-CONAPO-COLMEX, 2006. Conciliación de cifras sobre la dinámica demográfica de México.

INEGI, 2000. Modelo Digital de Elevación (grid 120m). http://www.inegi.org.mx/geo/contenidos/datosrelieve/continental/descarga.aspx. INEGI, Aguascalientes.

INEGI, 2001. Conjunto de Datos Vectoriales de la Carta de Uso de Suelo y Vegetación, 1:250,000, (serie II). Instituto Nacional de Estadística, Geografía e Informática, Aguascalientes

INEGI, 2002. Conjunto de Datos Edafológico Vectoriales (1:250,000). In: http://www.inegi.org.mx/geo/contenidos/recnat/edafologia/cartaedafologica.aspx (Ed.). INEGI, Aguascalientes.

INEGI, 2005. Conjunto de Datos Vectoriales de Uso del suelo y Vegetación, escala 1:250,000 (serie III) Instituto Nacional de Estadística, Geografía e Informática, Aguascalientes.

INEGI, 2008. Conjunto de Datos Vectoriales de Uso del suelo y Vegetación, escala 1:250,000 (serie IV) (preliminar version). Instituto Nacional de Estadística, Geografía e Informática, Aguascalientes.

Maderey, R., Torres-Ruata, C., 1990. Hidrografía e hidrometría. In: II, I.A.V. (Ed.), Atlas Nacional de México. Instituto de Geografía, UNAM, México, pp. 1:4,000,000.

Mendoza-Ponce, A., 2016. Vulnerability of Biodiversity to Land Use Cover Change and Climate Change in Mexico. University of Edinburgh.

Metzger, M., Bunce, R., Jongman, R., Sayre, R., Trabucco, A., Zomer, R., 2013. A high-resolution bioclimate map of the world: a unifying framework for global biodiversity research and monitoring. Global Ecology and Biogeography 22, 630-638.

Table S2. Relative Weights of Evidence on the 10 most significant variables per transition from natural covers to agriculture.

Transition:	Temperate Forest to Agriculture			Variable:	Aridity_index	
		Possible	Executed	Weight		
Range		Transitions	Transitions	Coefficient	Contrast	Significant?
0	5000	153632	3753	-0.30946906	-0.51400345	yes
5000	10000	159502	6195	0.16910379	0.34809976	yes
10000	15000	19549	960	0.41441172	0.44599021	yes
15000	30000	4081	204	0.43310678	0.43965913	yes
		336764	11112			
Transition:	Temperate Forest to Agriculture			Variable:	Protected_Areas_distance	
		Possible	Executed	Weight		
Range		Transitions	Transitions	Coefficient	Contrast	Significant?
0	1000	6718	303	0.32514225	0.33289318	yes
1000	11000	18641	1008	0.51599974	0.55542611	yes
11000	37000	60465	2728	0.32547425	0.41201516	yes
37000	38000	2416	80	0.00363501	0.00366132	no
38000	723000	248524	6993	-0.16428446	-0.52544236	yes
		336764	11112			
Transition:	Temperate Forest to Agriculture			Variable:	distance_cities	
		Possible	Executed	Weight		
Range		Transitions	Transitions	Coefficient	Contrast	Significant?
0	2000	3386	406	1.38941889	1.41765915	yes
2000	3000	6910	593	1.01693902	1.05250295	yes
3000	6000	30926	2072	0.74901001	0.86384625	yes
6000	8000	23075	1212	0.49022049	0.53686468	yes
8000	11000	34618	1486	0.27833268	0.31539421	yes
11000	12000	9557	332	0.05820681	0.05995715	no
12000	19000	68668	1968	-0.14044284	-0.17366957	yes
19000	24000	37375	858	-0.36818477	-0.40638341	yes
24000	43000	87843	1650	-0.57306937	-0.71906491	yes
43000	44000	2892	40	-0.88415201	-0.88932865	yes
44000	45000	2509	52	-0.47270834	-0.47557032	yes
45000	46000	2295	100	-0.75641426	-0.7601173	yes
46000	282000	26710	343	-0.94656865	-0.99841377	yes
		336764	11112			
Transition:	Temperate Forest to Agriculture			Variable:	distance_roads	
		Possible	Executed	Weight		

Range		Transitions	Transitions	Coefficient	Contrast	Significant?
0	1000	15293	1244	0.9535845	1.02821352	yes
1000	2000	32615	2011	0.65530511	0.75625515	yes
2000	3000	33300	1619	0.40389526	0.45901798	yes
3000	5000	46682	1839	0.18385817	0.21662222	yes
5000	6000	27649	888	-0.02792542	-0.03038808	no
6000	7000	18318	476	-0.24608893	-0.25865442	yes
7000	14000	96997	2096	-0.43499976	-0.57046505	yes
14000	16000	15883	270	-0.6796335	-0.70416661	yes
16000	34000	49085	664	-0.91160301	-1.01096621	yes
34000	35000	313	3	-1.26015629	-1.26083866	yes
35000	36000	206	1	-1.94520626	-1.94574597	no
36000	276000	423	1	-2.66720159	-2.6684083	yes
		336764	11112			
Transition:	Temperate Forest to Agriculture			Variable:	incomes	
		Possible	Executed	Weight		
Range		Transitions	Transitions	Coefficient	Contrast	Significant?
0	10000	143769	6156	-0.43768379	-0.89978211	yes
10000	20000	158761	3405	0.19957694	0.26886615	yes
20000	30000	27577	1167	0.70812608	0.85362685	yes
30000	40000	6657	384	1.03406501	1.08686729	yes
		336764	11112			
Transition:	Temperate Forest to Agriculture			Variable:	altitude	
		Possible	Executed	Weight		
Range		Transitions	Transitions	Coefficient	Contrast	Significant?
0	100	849	470	3.59299407	3.63504673	yes
100	200	298	63	2.06134679	2.06631058	yes
200	400	2144	143	0.73923988	0.74602878	yes
400	500	2254	101	0.3183006	0.32079809	yes
500	600	3494	166	0.37965857	0.38443794	yes
600	2000	174130	5967	0.03910812	0.08262809	yes
2000	2500	111005	3158	-0.15297706	-0.2208586	yes
2500	2800	32617	796	-0.3320731	-0.36221537	yes
2800	4300	9973	248	-0.29102317	-0.29876414	yes
		336764	11112			
Transition:	Temperate Forest to Agriculture			Variable:	potential_evapotranspiration	
		Possible	Executed	Weight		
Range		Transitions	Transitions	Coefficient	Contrast	Significant?

0	1000	139	27	1.95514172	1.95723049	yes
1000	2000	30178	1049	0.05390681	0.05936254	no
2000	3000	106843	6305	0.60861093	1.07734096	yes
3000	4000	102398	2299	-0.39588173	-0.53135819	yes
4000	5000	74494	1304	-0.64981862	-0.77955997	yes
5000	6000	22200	119	-1.84554558	-1.90499248	yes
6000	7000	512	9	-0.64556187	-0.6462974	no
		336764	11112			
Transition:	Temperate Forest to Agriculture			Variable:	population_density	
		Possible	Executed	Weight		
Range		Transitions	Transitions	Coefficient	Contrast	Significant?
0	100	314565	9568	-0.08407402	-0.86829677	yes
100	200	14657	1001	0.76462424	0.81618694	yes
200	14100	7542	543	0.82139048	0.84976431	yes
		336764	11112			
Transition:	Temperate Forest to Agriculture			Variable:	Soils	
		Possible	Executed	Weight		
Range		Transitions	Transitions	Coefficient	Contrast	Significant?
	Solonchak	14	11	4.67708671	4.6780679	yes
	Vertisol	3261	437	1.51182732	1.54323871	yes
	Litosol	92301	1501	-0.72472401	-0.90648048	yes
	Regosol	92171	2953	-0.03045749	-0.04171372	no
	Fluvisol	51	14	2.40594314	2.40709021	yes
	Tfeozem	50231	1661	0.0022175	0.00260671	no
	Xerosol	1535	58	0.14047845	0.14116587	no
	Yermosol	35	2	0.57444334	0.574522	no
	Planosol	951	68	0.81398623	0.81740937	yes
	Cambisol	36689	1319	0.08881359	0.10019489	yes
	Rendzina	7069	327	0.351652	0.36060093	yes
	Castagnozem	1022	39	0.15075625	0.15124902	no
	Acrisol	12615	431	0.03603292	0.03746001	no
	Luvisol	25754	1199	0.3583761	0.39415782	yes
	Gleysol	214	139	3.99478954	4.0071471	yes
	Chernozem	11	0	0	0	~
	Andosol	12307	814	0.7810466	0.82300196	yes
	Nitosol	171	36	2.05604788	2.05887824	yes
	Solonetz	339	31	1.08169114	1.08353858	yes
	Ranker	23	72	3	-1.75209499	yes
		336764	11112			

Transition:	Temperate Forest to Agriculture			Variable:	slope	
		Possible	Executed	Weight		
Range		Transitions	Transitions	Coefficient	Contrast	Significant?
0	2	3073	718	2.189965	2.24950388	yes
2	4	7855	866	1.28958358	1.34902633	yes
4	6	13882	951	0.76792269	0.81687302	yes
6	10	46423	2299	0.42326183	0.50946019	yes
10	12	30983	1135	0.10830625	0.11991592	yes
12	14	34721	1047	-0.09300566	-0.1031964	yes
14	18	71890	1726	-0.32723733	-0.4010882	yes
18	26	97316	1911	-0.53271288	-0.69068122	yes
26	30	18707	298	-0.74571003	-0.77671711	yes
30	58	11914	161	-0.91232761	-0.93447854	yes
		336764	11112			
Transition:	Temperate Forest to Agriculture			Variable:	seasonality	
		Possible	Executed	Weight		
Range		Transitions	Transitions	Coefficient	Contrast	Significant?
0	1000	10412	517	0.42606174	0.44284876	yes
1000	4000	228117	9069	0.19337484	0.77027284	yes
4000	5000	43862	948	-0.43479517	-0.48693028	yes
5000	6000	28734	405	-0.86995051	-0.92383287	yes
6000	7000	25553	172	-1.61645794	-1.68200206	yes
7000	8000	86	1	-1.06484754	-1.06501859	no
		336764	11112			
Transition:	Scrublands to Agriculture			Variable:	Index_marginalization	
		Possible	Executed	Weight		
Range		Transitions	Transitions	Coefficient	Contrast	Significant?
	1	271816	3742	-0.33660869	-0.58581339	yes
	2	160411	3253	0.05736013	0.08197122	yes
	3	58963	1945	0.55692912	0.64926826	yes
	4	57920	1590	0.36754091	0.42079257	yes
	5	228	1	-1.43094187	-1.43124416	no
		549338	10531			
Transition:	Scrublands to Agriculture			Variable:	Aridity_index	
		Possible	Executed	Weight		
Range		Transitions	Transitions	Coefficient	Contrast	Significant?
0	5000	548784	10500	-0.0019769	-1.11141679	yes

5000	10000	554	31	1.10943989	1.11141679	yes
		549338	10531			
Transition:	Scrublands to Agriculture			Variable:	Protected_Areas_distance	
		Possible	Executed	Weight		
Range		Transitions	Transitions	Coefficient	Contrast	Significant?
0	1000	5334	58	-0.57544636	-0.57976384	yes
1000	2000	1685	55	0.54603204	0.54823861	yes
2000	245000	341758	9155	0.34238474	1.41703582	yes
245000	246000	1144	5	-1.4934339	-1.49507517	yes
246000	247000	1193	8	-1.06302236	-1.06446414	yes
247000	249000	2182	25	-0.52256367	-0.52419823	yes
249000	265000	16630	154	-0.73767331	-0.75399779	yes
265000	266000	1026	7	-1.04563274	-1.04686082	yes
266000	299000	34748	173	-1.36256041	-1.41231725	yes
299000	300000	1022	1	-2.99350367	-2.99530543	yes
300000	311000	11266	30	-1.99064666	-2.00886784	yes
311000	312000	945	6	-1.11802186	-1.11919621	yes
312000	316000	3811	17	-1.47292866	-1.47837946	yes
316000	317000	947	2	-2.2230036	-2.22456908	yes
317000	358000	37943	147	-1.61449182	-1.67316407	yes
358000	359000	922	11	-0.48161348	-0.4822606	no
359000	360000	922	4	-1.50086888	-1.5021942	yes
360000	361000	928	6	-1.09975161	-1.10089435	yes
361000	363000	1828	8	-1.49211609	-1.49473969	yes
363000	367000	3520	22	-1.13387005	-1.13829208	yes
367000	372000	4198	36	-0.81519792	-0.81952805	yes
372000	373000	809	11	-0.34917918	-0.34961624	no
373000	376000	2355	23	-0.68395318	-0.68610424	yes
376000	391000	10885	132	-0.46510399	-0.47264924	yes
391000	392000	660	11	-0.1425033	-0.14266345	no
392000	396000	2501	30	-0.47614668	-0.47789048	yes
396000	397000	608	3	-1.37158202	-1.37242059	yes
397000	398000	607	1	-2.47184584	-2.47287622	yes
398000	399000	652	4	-1.15256219	-1.15338566	yes
399000	400000	629	2	-1.81276521	-1.81373964	yes
400000	404000	2567	13	-1.34543253	-1.34894869	yes
404000	405000	658	2	-1.85797946	-1.85900777	yes
405000	406000	656	5	-0.93403758	-0.93477164	yes
406000	411000	3150	17	-1.2814988	-1.28571488	yes
411000	413000	1212	4	-1.77539287	-1.77725747	yes

413000	415000	1259	2	-2.50830188	-2.5104476	yes
415000	558000	35176	115	-1.7848784	-1.84118346	yes
558000	723000	11002	226	-0.00977868	-0.00978148	no
		549338	10531			
Transition:	Scrublands to Agriculture			Variable:	incomes_density	
		Possible	Executed	Weight		
Range		Transitions	Transitions	Coefficient	Contrast	Significant?
0	2000	73369	859	-0.40515353	-0.46404432	yes
2000	3000	58435	387	-0.98004353	-1.06426909	yes
3000	4000	51212	450	-0.69509843	-0.75389115	yes
4000	5000	32464	1598	1.0696549	1.21391692	yes
5000	6000	27906	670	0.32554011	0.34945468	yes
6000	7000	17308	512	0.53998589	0.56683602	yes
7000	8000	43548	558	-0.31380654	-0.34051747	yes
8000	9000	52679	384	-0.88345618	-0.95421314	yes
9000	10000	77860	304	0.85250348	0.87391455	yes
10000	11000	9628	534	1.19558301	1.24206503	yes
11000	12000	17026	824	-1.09086927	-1.11533549	yes
12000	13000	13926	471	0.67830934	0.70728351	yes
13000	15000	10693	195	0.04461682	0.04565816	no
15000	16000	3824	816	0.33872523	0.34195147	yes
16000	19000	12855	224	-0.00170603	-0.00175303	no
19000	20000	4697	216	0.9982342	1.01483968	yes
20000	21000	8589	764	-1.30932324	-1.32273453	yes
21000	22000	15963	301	0.0786749	0.08149606	no
22000	23000	7056	70	-0.57261088	-0.57915689	yes
23000	24000	1135	36	0.61192029	0.6138973	yes
24000	25000	1406	69	1.06648023	1.07192169	yes
25000	26000	1422	39	0.46210862	0.46384109	yes
26000	30000	4195	214	1.107245	1.12467684	yes
30000	31000	1897	33	-0.00341513	-0.00342868	no
31000	32000	242	3	-0.34729396	-0.34744299	no
32000	33000	3	0	0	0	~
		549338	10531			
Transition:	Scrublands to Agriculture			Variable:	distance_cities	
		Possible	Executed	Weight		
Range		Transitions	Transitions	Coefficient	Contrast	Significant?
0	2000	2809	159	1.1239994	1.13432783	yes
2000	8000	37282	1608	0.83797916	0.93569036	yes

8000	9000	9084	304	0.57420613	0.58715072	yes
9000	20000	109792	3006	0.36719358	0.48348539	yes
20000	21000	10009	229	0.18303736	0.18676076	yes
21000	45000	203042	3905	0.00567487	0.00902145	no
45000	46000	6226	88	-0.3075073	-0.31055573	yes
46000	52000	33676	378	-0.5409483	-0.56812177	yes
52000	53000	4491	20	-1.47222499	-1.47865762	yes
53000	71000	64163	417	-1.09216538	-1.17765852	yes
71000	72000	2685	44	-0.15731316	-0.15803053	no
72000	78000	15085	189	-0.42969087	-0.43958167	yes
78000	79000	2046	16	-0.90579223	-0.90804477	yes
79000	80000	2497	41	-1.71457768	-1.71762143	yes
80000	287000	46451	127	-1.96181826	-2.039624	yes
		549338	10531			
Transition:	Scrublands to Agriculture			Variable:	distance_roads	
		Possible	Executed	Weight		
Range		Transitions	Transitions	Coefficient	Contrast	Significant?
0	1000	24193	1006	0.79742444	0.85384043	yes
1000	2000	48603	1573	0.53723308	0.6076773	yes
2000	3000	46846	1246	0.33506485	0.37255947	yes
3000	6000	103037	2250	0.13295499	0.16622167	yes
6000	7000	25719	467	-0.05529723	-0.05793895	no
7000	16000	170227	2576	-0.24061248	-0.33282483	yes
16000	17000	10354	103	-0.6653674	-0.67474729	yes
17000	19000	19388	251	-0.39889183	-0.41093224	yes
19000	54000	96879	1034	-0.59426338	-0.6867895	yes
54000	56000	630	14	0.15084451	0.15103089	no
56000	57000	221	8	0.65318352	0.65354808	no
57000	58000	200	1	-1.35827068	-1.35854512	no
58000	62000	656	2	-1.85492602	-1.85595062	yes
62000	63000	141	0	0	0	~
63000	279000	2244	0	0	0	~
		549338	10531			
Transition:	Scrublands to Agriculture			Variable:	altitude	
		Possible	Executed	Weight		
Range		Transitions	Transitions	Coefficient	Contrast	Significant?
0	100	40651	1035	0.2901097	0.31718631	yes
100	200	41458	881	0.10504229	0.11410449	yes
200	300	37302	582	-0.20966505	-0.22340462	yes

300	400	29999	355	-0.48995589	-0.51225924	yes
400	500	27699	240	-0.80486899	-0.83412748	yes
500	700	45209	496	-0.56650229	-0.60489804	yes
700	800	17806	378	0.10400215	0.1076729	yes
800	900	17170	441	0.2990872	0.31032225	yes
900	1000	15720	218	-0.32928792	-0.33756558	yes
1000	1100	20487	215	-0.61141653	-0.62914286	yes
1100	1200	29569	382	-0.40111673	-0.41986602	yes
1200	1500	108200	1674	-0.21823177	-0.26542105	yes
1500	1600	22060	867	0.73855411	0.78433831	yes
1600	1700	16773	511	0.47472456	0.49381553	yes
1700	1800	16164	304	-0.01958645	-0.02017457	no
1800	1900	15396	375	0.24466287	0.2526446	yes
1900	2000	15105	518	0.59703059	0.62002105	yes
2000	3200	32570	1059	0.54418545	0.59004008	yes
		549338	10531			
Transition:	Scrublands to Agriculture			Variable:	Potential_evapotranspiration	
		Possible	Executed	Weight		
Range		Transitions	Transitions	Coefficient	Contrast	Significant?
0	1000	10829	128	-0.49102806	-0.49885942	yes
1000	3000	7058	380	1.06863158	1.09291115	yes
3000	4000	52878	1885	0.6372736	0.73507685	yes
4000	5000	164299	2363	-0.29223505	-0.39559032	yes
5000	6000	265635	5337	0.04787072	0.09478283	yes
6000	7000	47714	396	-0.84819769	-0.90178706	yes
7000	8000	925	42	0.88937857	0.89173461	yes
		549338	10531			
Transition:	Scrublands to Agriculture			Variable:	slope	
		Possible	Executed	Weight		
Range		Transitions	Transitions	Coefficient	Contrast	Significant?
0	2	258135	7970	0.48838662	1.27817189	yes
2	4	77964	1364	-0.09335269	-0.10802071	yes
4	6	43594	466	-0.59291925	-0.63111118	yes
6	8	33287	236	-1.00715247	-1.04780846	yes
8	14	71584	360	-1.35265826	-1.45969937	yes
14	16	16617	44	-1.99651768	-2.02358178	yes
16	48	48157	91	-2.34225916	-2.42681008	yes
		549338	10531			

Transition:	Scrublands to Agriculture			Variable:	seasonality	
		Possible	Executed	Weight		
Range		Transitions	Transitions	Coefficient	Contrast	Significant?
0	1000	10829	128	-0.49102806	-0.49885942	yes
1000	2000	3425	181	1.04896876	1.06026663	yes
2000	4000	108229	2849	0.32442934	0.42223865	yes
4000	5000	110252	1264	-0.52192234	-0.620052	yes
5000	6000	139446	4053	0.42630984	0.6228272	yes
6000	7000	123307	1660	-0.35927166	-0.44362349	yes
7000	8000	49362	383	-0.91607778	-0.97433426	yes
8000	9000	4488	13	-1.90627813	-1.91338298	yes
		549338	10531			
Transition:	Scrublands to Agriculture			Variable:	Soils	
		Possible	Executed	Weight		
Range		Transitions	Transitions	Coefficient	Contrast	Significant?
	Solonchak	7655	287	0.68961479	0.70347689	yes
	Vertisol	17590	727	0.79108347	0.83081916	yes
	Litosol	126485	665	-1.3077864	-1.50849894	yes
	Regosol	149777	1600	-0.59336973	-0.75017143	yes
	Fluvisol	5110	42	-0.85799778	-0.86345206	yes
	Tfeozem	12181	517	0.81881457	0.84726817	yes
	Xerosol	135259	4566	0.58082063	0.87142398	yes
	Yermosol	57416	1060	-0.03838566	-0.04277494	no
	Planosol	925	49	1.05148836	1.05452501	yes
	Cambisol	2324	83	0.63919728	0.64294214	yes
	Rendzina	27500	515	-0.02383539	-0.02507644	no
	Castagnozem	5356	309	1.14182614	1.16219603	yes
	Acrisol	9	0	0	0	~
	Luvisol	552	18	0.54501007	0.5457292	yes
	Gleysol	268	35	2.03934376	2.04224028	yes
	Chernozem	72	0	0	0	~
	Andosol	83	8	1.69698758	1.69760832	yes
	Nitosol	0	0	0	0	~
	Solonetz	0	0	0	0	~
	Ranker	776	50	1.33007946	1.33351199	yes
		549338	10531			
Transition:	Tropical Evergreen Forests to Agriculture			Variable:	Index_marginalization	
		Possible	Executed	Weight		
Range		Transitions	Transitions	Coefficient	Contrast	Significant?

	1	4355	74	-1.44152891	-1.47790022	yes
	2	14956	902	-0.12969991	-0.15125437	yes
	3	10949	947	0.25910664	0.29561781	yes
	4	49868	3053	-0.11373105	-0.21820358	yes
	5	19147	1784	0.34121005	0.43982836	yes
		99275	6760			
Transition:	Tropical Evergreen Forests to Agriculture			Variable:	Aridity_index	
		Possible	Executed	Weight		
Range		Transitions	Transitions	Coefficient	Contrast	Significant?
0	5000	1962	90	-0.41860508	-0.42564413	yes
5000	15000	82500	5278	-0.06678925	-0.34913447	yes
15000	20000	10223	1099	0.49984027	0.57343221	yes
20000	25000	4262	255	-0.13818666	-0.14401273	yes
25000	30000	308	38	0.6555121	0.65822655	yes
30000	35000	20	0	~	0	~
		99275	6760			
Transition:	Tropical Evergreen Forests to Agriculture			Variable:	Protected_Areas_distance	
		Possible	Executed	Weight		
Range		Transitions	Transitions	Coefficient	Contrast	Significant?
0	1000	4805	55	-1.84221881	-1.88675744	yes
1000	2000	741	57	0.13144125	0.13248809	no
2000	5000	2143	132	-0.10723756	-0.10949452	no
5000	7000	1650	125	0.11491195	0.11695499	no
7000	40000	32498	2858	0.27734518	0.44065667	yes
40000	41000	994	114	0.57262444	0.58007467	yes
41000	47000	6685	635	0.36215935	0.3931724	yes
47000	48000	1130	87	0.13239957	0.13401504	no
48000	79000	30246	1978	-0.04329629	-0.06176803	yes
79000	80000	655	21	-0.79117861	-0.7949438	yes
80000	81000	697	35	-0.32356959	-0.32555995	no
81000	82000	648	23	-0.68590953	-0.68927995	yes
82000	178000	16383	640	-0.58633502	-0.67340503	yes
		99275	6760			
Transition:	Tropical Evergreen Forests to Agriculture			Variable:	distance_cities	
		Possible	Executed	Weight		
Range		Transitions	Transitions	Coefficient	Contrast	Significant?
0	2000	636	92	0.84065617	0.84849018	yes
2000	7000	8582	1022	0.61670714	0.69572596	yes

7000	8000	2796	266	0.36533856	0.37782874	yes
8000	26000	45828	3674	0.17776824	0.35588875	yes
26000	27000	1948	99	-0.30946355	-0.31488258	yes
27000	77000	37577	1588	-0.50292114	-0.72772188	yes
77000	78000	142	0	0	0	~
78000	79000	121	1	-2.16967491	-2.17082589	yes
79000	80000	118	0	0	0	~
80000	81000	116	18	-2.12711529	-2.1282121	yes
81000	82000	220	0	0	0	~
82000	106000	1191	0	0	0	~
		99275	6760			
Transition:	Tropical Evergreen Forests to Agriculture			Variable:	distance_rivers	
		Possible	Executed	Weight		
Range		Transitions	Transitions	Coefficient	Contrast	Significant?
0	1000	3350	333	0.41247215	0.42983266	yes
1000	56000	72716	6111	0.22765868	1.29826233	yes
56000	57000	220	2	-2.07499998	-2.07706323	yes
57000	58000	228	5	-1.18138596	-1.18305937	yes
58000	59000	212	2	-2.03761245	-2.03958903	yes
59000	60000	214	4	-1.34446527	-1.34614586	yes
60000	61000	230	2	-2.11985054	-2.12202215	yes
61000	62000	233	4	-1.43107974	-1.43296619	yes
62000	64000	447	4	-2.09092751	-2.09513553	yes
64000	67000	627	9	-1.61291598	-1.61828614	yes
67000	88000	3281	28	-2.13878051	-2.17042483	yes
88000	89000	135	2	-1.58085404	-1.58199678	yes
89000	90000	141	0	0	0	~
90000	91000	131	1	-2.25118655	-2.25244477	yes
91000	92000	133	0	0	0	~
92000	93000	129	3	-1.12132171	-1.1222407	no
93000	94000	122	2	-1.47799666	-1.47899869	yes
94000	95000	123	1	-2.18767314	-2.18884478	yes
95000	96000	118	2	-1.44409511	-1.44505384	yes
96000	99000	370	3	-2.19040166	-2.19393258	yes
99000	100000	114	0	0	0	~
100000	116000	1547	13	-2.15433672	-2.16913189	yes
116000	117000	98	2	-1.25485311	-1.25559541	no
117000	118000	96	0	0	0	~
118000	146000	2723	16	-2.51465966	-2.54198673	yes
146000	147000	101	4	-0.57206871	-0.57252585	no

147000	148000	96	3	-0.8176393	-0.81820116	no
148000	150000	210	5	-1.09722416	-1.09870256	yes
150000	151000	101	0	0	0	~
151000	154000	304	4	-1.70114021	-1.70379631	yes
154000	155000	94	5	-0.26285055	-0.2630731	no
155000	158000	276	10	-0.66456331	-0.66596228	yes
158000	159000	86	1	-1.82630335	-1.82707461	no
159000	160000	89	2	-1.15641303	-1.15705796	no
160000	161000	85	1	-1.8144689	-1.81522933	no
161000	162000	90	2	-1.16784173	-1.16849748	no
162000	163000	82	4	-0.35406656	-0.35431813	no
163000	164000	79	1	-1.74036092	-1.74105645	no
164000	165000	76	2	-0.99457001	-0.9950743	no
165000	166000	79	4	-0.31484585	-0.31506497	no
166000	167000	75	3	-0.56170593	-0.5620406	no
167000	168000	69	2	-0.89519754	-0.8956261	no
168000	184000	978	22	-1.15536756	-1.16249503	yes
184000	187000	142	2	-1.63214734	-1.63336585	yes
187000	190000	153	1	-2.40753262	-2.40902901	yes
190000	191000	55	0	0	0	~
191000	198000	394	3	-2.25374737	-2.25753878	yes
198000	199000	53	0	0	0	~
199000	200000	63	1	-1.51078648	-1.51130893	no
200000	201000	62	0	0	0	~
201000	202000	57	8	0.80396915	0.8046235	yes
202000	203000	56	10	1.0902916	1.09127464	yes
203000	204000	64	5	0.14824837	0.14835035	no
204000	205000	59	3	-0.3103915	-0.3105531	no
205000	206000	58	4	0.01365822	0.01366625	no
206000	207000	58	5	0.2554939	0.25566078	no
207000	208000	61	3	-0.34548282	-0.34566605	no
208000	209000	62	1	-1.49452596	-1.49503759	no
209000	210000	64	2	-0.8176393	-0.81801379	no
210000	211000	62	1	-1.49452596	-1.49503759	no
211000	212000	65	4	-0.1082316	-0.10829928	no
212000	213000	64	3	-0.39591367	-0.39612936	no
213000	214000	63	0	0	0	~
214000	216000	120	1	-2.16277559	-2.16391476	yes
216000	217000	66	0	0	0	~
217000	219000	122	1	-2.17944264	-2.18060345	yes
219000	220000	60	2	-0.75094793	-0.75127915	no

220000	221000	62	0	0	0	~
221000	247000	1562	10	-2.4283667	-2.44380428	yes
247000	248000	69	7	0.43512367	0.43548932	no
248000	249000	66	9	0.77052121	0.77123715	yes
249000	250000	65	6	0.33056993	0.33081996	no
250000	256000	406	28	0.01365822	0.01371464	no
256000	257000	64	0	0	0	~
257000	346000	4190	16	-2.94769346	-2.99149026	yes
		99275	6760			
Transition:	Tropical Evergreen Forests to Agriculture			Variable:	distance_roads	
		Possible	Executed	Weight		
Range		Transitions	Transitions	Coefficient	Contrast	Significant?
0	1000	4290	661	0.91338934	0.97627126	yes
1000	2000	10046	1110	0.53061986	0.6084087	yes
2000	6000	31573	2848	0.30519707	0.48039686	yes
6000	7000	5243	390	0.09514229	0.10068343	no
7000	8000	5569	342	-0.11043414	-0.11667559	yes
8000	10000	9221	481	-0.2834503	-0.3088743	yes
10000	13000	9370	377	-0.55560868	-0.60048472	yes
13000	14000	2693	81	-0.85707443	-0.8736593	yes
14000	35000	20315	466	-1.13537537	-1.30544647	yes
35000	36000	235	1	-2.83897321	-2.8413578	yes
36000	37000	194	2	-1.94800029	-1.94978188	yes
37000	38000	144	0	0	0	~
38000	39000	128	1	-2.22783918	-2.22906494	yes
39000	40000	100	0	0	0	~
40000	47000	154	0	0	0	~
		99275	6760			
Transition:	Tropical Evergreen Forests to Agriculture			Variable:	Potential_evapotranspiration	
		Possible	Executed	Weight		
Range		Transitions	Transitions	Coefficient	Contrast	Significant?
0	1000	1472	70	-0.38081192	-0.38567321	yes
1000	2000	2461	91	-0.64343782	-0.65583617	yes
2000	3000	80516	5098	-0.07784976	-0.36331635	yes
3000	4000	14815	1501	0.43366334	0.52936347	yes
4000	5000	11	0	0	0	~
		99275	6760			
Transition:	Tropical Evergreen Forests to Agriculture			Variable:	GDP	

		Possible	Executed	Weight		
Range		Transitions	Transitions	Coefficient	Contrast	Significant?
0	100	1285	61	-0.37184564	-0.37617565	yes
100	300	9745	624	-0.0550244	-0.06113299	no
300	400	1449	153	0.49056	0.49998104	yes
400	500	2065	181	0.28450453	0.29166237	yes
500	600	3099	509	1.00019482	1.05320319	yes
600	700	3444	211	-0.10214769	-0.10580915	no
700	900	3877	289	0.10823642	0.11306035	no
900	1000	1392	255	1.13227501	1.15992074	yes
1000	1400	2648	327	0.66736672	0.69316436	yes
1400	1500	1464	109	0.1069511	0.10870142	no
1500	1600	1084	49	-0.42317645	-0.42723638	yes
1600	1700	10228	127	-1.74904267	-1.84979409	yes
1700	1800	816	56	0.01919321	0.01936048	no
1800	1900	1380	169	0.65785693	0.67080501	yes
1900	2300	1833	522	1.70628206	1.77621616	yes
2300	2400	172	3	-1.40412647	-1.40556367	yes
2400	2500	243	26	0.50535914	0.50697099	yes
2500	2600	5734	321	-0.19795767	-0.20954618	yes
2600	2700	251	18	0.06649326	0.06667412	no
2700	2800	1452	256	1.08559947	1.11273429	yes
2800	2900	55	4	0.08162868	0.08167755	no
2900	3000	11931	93	-2.21931053	-2.34776401	yes
3000	3100	154	30	1.20807577	1.21136252	yes
3100	3200	25	7	1.68269835	1.6835866	yes
3200	3400	8	0	0	0	~
3400	3500	271	117	2.35238129	2.36903829	yes
3500	3600	492	51	0.46994071	0.47293873	yes
3600	4000	105	36	1.97657239	1.98141847	yes
4000	4300	1	0	0	0	~
4300	4600	121	12	0.42071872	0.42136163	no
4600	4700	128	7	-0.22272044	-0.22299278	no
4700	4900	449	84	1.1580794	1.16713182	yes
4900	5200	1	0	0	0	~
5200	5400	1667	50	-0.8491449	-0.85969999	yes
5400	5800	6	2	1.93401277	1.93427931	yes
5800	5900	72	5	0.03190525	0.03192972	no
5900	6500	3616	549	0.90680323	0.96105439	yes
6500	6700	28	0	0	0	~
6700	7100	2024	17	-2.14402305	-2.16422781	yes

7100	8300	158	19	0.637125	0.63852265	yes
8300	8600	122	12	0.41158624	0.41221788	no
8600	9100	3727	0	0	0	~
9100	9600	324	124	2.14912415	2.1663734	yes
9600	9700	3862	225	-0.15565409	-0.16178989	yes
9700	10600	385	43	0.55354933	0.55641164	yes
10600	12400	339	0	0	0	~
12400	14700	9782	425	-0.46463088	-0.50746287	yes
14700	15100	1680	9	-2.596793	-2.61438334	yes
15100	20800	4032	226	-0.19663909	-0.20459947	yes
20800	23100	47	346	0.70534736	0.70582064	no
23100	30100	2	1	2.62715996	2.62730447	no
		99275	6760			
Transition:	Tropical Evergreen Forests to Agriculture			Variable:	Slope	
		Possible	Executed	Weight		
Range		Transitions	Transitions	Coefficient	Contrast	Significant?
0	2	52332	2893	-0.22258378	-0.42918095	yes
2	4	11788	1052	0.29295231	0.33871587	yes
4	6	4578	569	0.6634451	0.70706958	yes
6	14	15505	1520	0.39658679	0.48736119	yes
14	16	3487	226	-0.0533924	-0.05529202	no
16	20	5466	279	-0.30683721	-0.32242495	yes
20	24	3256	139	-0.49429057	-0.5078097	yes
24	26	981	34	-0.71107679	-0.71633082	yes
26	28	808	23	-0.93805669	-0.94268371	yes
28	48	1074	25	-1.12085501	-1.12856242	yes
		99275	6760			
Transition:	Tropical Evergreen Forests to Agriculture			Variable:	Soils	
		Possible	Executed	Weight		
Range		Transitions	Transitions	Coefficient	Contrast	Significant?
	Solonchak	1279	120	0.3485268	0.3538309	yes
	Vertisol	9521	1333	0.80111028	0.92807798	yes
	Litosol	15679	538	-0.72095511	-0.81674361	yes
	Regosol	13333	718	-0.24982439	-0.28413152	yes
	Fluvisol	10	0	0	0	~
	Tfeozem	2041	124	-0.12188727	-0.12431241	no
	Xerosol	0	0	0	0	~
	Yermosol	0	0	0	0	~
	Planosol	0	0	0	0	~

	Cambisol	4122	671	0.97869772	1.04522116	yes
	Rendzina	36000	1247	-0.71117733	-0.97830305	yes
	Castagnozem	0	0	0	0	~
	Acrisol	6211	752	0.63406333	0.6911751	yes
	Luvisol	5507	494	0.2990936	0.31926916	yes
	Gleysol	3181	414	0.7166949	0.74952804	yes
	Chernozem	0	0	0	0	~
	Andosol	24	0	0	0	~
	Nitosol	6	2	1.92320072	1.92345339	yes
	Solonetz	2361	347	0.87715383	0.90808016	yes
	Ranker	0	0	0	0	~
		99275	6760			
Transition:	Tropical Dry Forests to Agriculture			Variable:	Aridity_index	
		Possible	Executed	Weight		
Range		Transitions	Transitions	Coefficient	Contrast	Significant?
0	5000	130972	8426	-0.13477057	-0.31264966	yes
5000	10000	86441	7421	0.17700657	0.30997874	yes
10000	20000	385	39	0.35951661	0.36025953	yes
		217798	15886			
Transition:	Tropical Dry Forests to Agriculture			Variable:	distance_cities	
		Possible	Executed	Weight		
Range	Transitions	Transitions	Coefficient	Contrast	Significant?	
0	2000	3333	594	1.0174121	1.04203108	yes
2000	4000	14404	1990	0.71519072	0.7861773	yes
4000	6000	20437	2296	0.47887486	0.54152054	yes
6000	9000	30098	2857	0.29092988	0.34511316	yes
9000	17000	59870	4762	0.0972538	0.13642186	yes
17000	18000	6476	410	-0.14841658	-0.15268531	yes
18000	19000	5987	287	-0.44285829	-0.45321272	yes
19000	38000	61182	2405	-0.65031977	-0.82988636	yes
38000	39000	1518	18	-1.87696768	-1.88329502	yes
39000	40000	1313	24	-1.43768722	-1.44258089	yes
40000	133000	13180	243	-1.78335426	-1.83764401	yes
		217798	15886			
Transition:	Tropical Dry Forests to Agriculture			Variable:	distance_roads	
		Possible	Executed	Weight		
Range	Transitions	Transitions	Coefficient	Contrast	Significant?	
0	1000	14315	2281	0.87927171	0.97282235	yes

1000	2000	31705	3423	0.43068554	0.52245414	yes
2000	3000	29701	2638	0.21424731	0.25192972	yes
3000	6000	55198	4004	-0.00593474	-0.00794259	no
6000	7000	11652	620	-0.3364422	-0.35281912	yes
7000	18000	61674	2713	-0.53642743	-0.69448854	yes
18000	19000	2055	48	-1.1908016	-1.1977652	yes
19000	27000	8527	139	-1.55768972	-1.59133179	yes
27000	28000	458	2	-2.88695189	-2.88908695	yes
28000	30000	792	9	-1.92351438	-1.92683315	yes
30000	43000	1675	9	-2.6785625	-2.68628116	yes
43000	47000	46	0	0	0	~
		217798	15886			
Transition:	Tropical Dry Forests to Agriculture			Variable:	altitude	
		Possible	Executed	Weight		
Range	Transitions	Transitions	Coefficient	Contrast	Significant?	
0	100	41584	4021	0.30790836	0.3939217	yes
100	200	22843	1676	0.00636389	0.00711187	no
200	600	63734	3923	-0.18193567	-0.24961426	yes
600	2700	89637	6266	-0.0474015	-0.07936229	yes
		217798	15886			
Transition:	Tropical Dry Forests to Agriculture			Variable:	Potential_evapotranspiration	
		Possible	Executed	Weight		
Range	Transitions	Transitions	Coefficient	Contrast	Significant?	
0	1000	3760	459	0.56946322	0.58229839	yes
1000	2000	4391	220	-0.39988981	-0.40681821	yes
2000	3000	75527	8038	0.41460942	0.7129485	yes
3000	4000	84378	5990	-0.0291857	-0.04727461	yes
4000	5000	29725	1006	-0.80918311	-0.8971883	yes
5000	6000	19947	173	-2.19643789	-2.28855538	yes
6000	7000	70	0	0	0	~
		217798	15886			
Transition:	Tropical Dry Forests to Agriculture			Variable:	GDP	
		Possible	Executed	Weight		
Range	Transitions	Transitions	Coefficient	Contrast	Significant?	
0	100	26589	977	-0.71179779	-0.78529831	yes
100	200	24787	1290	-0.3476987	-0.38678542	yes
200	300	19340	1392	-0.00220529	-0.00242655	no
300	2500	106962	9216	0.19310066	0.42955089	yes

2500	2600	1160	212	1.0567636	1.06589919	yes
2600	2700	662	54	0.133341	0.13378354	no
2700	2800	497	58	0.53047543	0.53204249	yes
2800	3000	2842	95	-0.80985596	-0.81769013	yes
3000	3200	30	8	1.54293092	1.54334262	yes
3200	3300	1859	82	-0.52143075	-0.52512969	yes
3300	3400	150	15	0.35730726	0.35760238	no
3400	3500	318	69	1.27118544	1.27444328	yes
3500	3600	136	22	0.90937584	0.91023642	yes
3600	3700	362	27	0.03623817	0.03630115	no
3700	3800	209	21	0.36261231	0.36303071	no
3800	4000	152	30	1.15170817	1.15305239	yes
4000	4400	516	61	0.54510828	0.54679084	yes
4400	4500	81	7	0.19637689	0.19645859	no
4500	4800	536	60	0.48345854	0.48496835	yes
4800	5000	226	42	1.07726569	1.07908137	yes
5000	5100	49	6	0.58509119	0.58526514	no
5100	5400	5990	318	-0.32671385	-0.33495869	yes
5400	5500	1	0	0	0	~
5500	5800	260	43	0.93583459	0.93754788	yes
5800	6000	1158	81	-0.03295369	-0.03313222	no
6000	6100	47	7	0.81156253	0.8118172	yes
6100	6600	297	99	1.86138465	1.86687598	yes
6600	6700	818	30	-0.71376888	-0.71581976	yes
6700	6800	166	12	0.00248588	0.00248783	no
6800	6900	31	4	0.64498933	0.64511376	no
6900	7300	49	1	-1.31666918	-1.3168479	no
7300	7500	595	79	0.67787292	0.68042767	yes
7500	7600	166	8	-0.42862166	-0.42890197	no
7600	8100	4	0	0	0	~
8100	8600	1083	42	-0.65573562	-0.65829223	yes
8600	9000	103	2	-1.3674415	-1.36782444	no
9000	9300	758	100	0.67049709	0.67370964	yes
9300	9400	8	0	0	0	~
9400	9700	113	9	0.10736551	0.10742549	no
9700	10300	1330	37	-0.99927063	-1.00344456	yes
10300	11900	304	4	-1.76295628	-1.76422147	yes
11900	12400	917	154	0.9542264	0.96046594	yes
12400	13100	27	1	-0.70356471	-0.70363151	no
13100	14000	7	0	0	0	~
14000	14200	35	5	0.76277236	0.76294699	no

14200	14400	11	2	1.05045444	1.05053953	no
14400	14700	364	29	0.10769713	0.10789122	no
14700	15100	55	0	0	0	~
15100	15300	102	15	0.79667391	0.7972133	yes
15300	15700	3	1	1.86138465	1.86143991	no
15700	16400	1627	15	-2.12264889	-2.12989927	yes
16400	19300	1	0	0	0	~
19300	20800	3199	126	-0.63959582	-0.64706975	yes
20800	21300	396	24	-0.18630819	-0.18663035	no
21300	22800	461	108	1.370195	1.37548981	yes
22800	26600	2477	76	-0.89837542	-0.90567672	yes
26600	26900	72	1	-1.70814804	-1.70844378	no
26900	29100	78	12	0.84978374	0.85023351	yes
29100	30900	7217	695	-0.03844904	-0.03868646	no
30900	32600	5	2	2.14906672	2.14918234	yes
		217798	15886			
Transition:	Tropical Dry Forests to Agriculture			Variable:	Population	
		Possible	Executed	Weight		
Range	Transitions	Transitions	Coefficient	Contrast	Significant?	
0	10000	67749	3165	-0.24028376	-0.4219821	yes
10000	20000	31051	2629	0.39496994	0.53493623	yes
20000	30000	37837	2295	0.03554764	0.04872137	no
30000	40000	5519	250	-0.27260506	-0.28233769	yes
		142156	8339			
Transition:	Tropical Dry Forests to Agriculture			Variable:	Slope	
		Possible	Executed	Weight		
Range	Transitions	Transitions	Coefficient	Contrast	Significant?	
0	2	45764	4164	0.24133793	0.31483676	yes
2	4	19777	2311	0.52038614	0.58724452	yes
4	10	49366	4744	0.30161559	0.406887	yes
10	12	16941	1084	-0.1399912	-0.15106779	yes
12	16	32623	1713	-0.34987159	-0.40188266	yes
16	20	25934	1087	-0.58635347	-0.64677334	yes
20	24	15487	531	-0.79514387	-0.83810291	yes
24	26	4408	113	-1.09485707	-1.10922103	yes
26	32	5845	103	-1.47787193	-1.50022231	yes
32	54	1653	36	-1.75270246	-1.75904262	yes
		217798	15886			

Transition:	Tropical Dry Forests to Agriculture			Variable:	Soils	
		Possible	Executed	Weight		
Range	Transitions	Transitions	Coefficient	Contrast	Significant?	
	Solonchak	1112	67	-0.2046858	-0.2056483	no
	Vertisol	11479	1685	0.78238935	0.84479299	yes
	Litosol	48196	1830	-0.68985672	-0.82835831	yes
	Regosol	65332	4493	-0.06331666	-0.08943205	yes
	Fluvisol	362	26	-0.01662088	-0.01664836	no
	Tfeozem	23528	2154	0.24754509	0.28136375	yes
	Xerosol	1393	158	0.48616252	0.49002287	yes
	Yermosol	561	60	0.4201322	0.4214319	yes
	Planosol	24708	2453	0.3371388	0.38808017	yes
	Cambisol	24495	1660	-0.0790831	-0.08873224	yes
	Rendzina	885	64	-0.00924629	-0.00928386	no
	Castagnozem	1457	182	0.59569897	0.60088708	yes
	Acrisol	13427	1017	0.04074826	0.04347559	no
	Luvisol	115	7	-0.19382734	-0.19392163	no
	Gleysol	25	0	0	0	~
	Chernozem	712	29	0.37743003	0.37784607	no
	Andosol	0	0	0	0	~
	Nitosol	11	1	0.23980865	0.23982207	no
	Solonetz	0	0	0	0	~
		217798	15886			
Transition:	Tropical Dry Forests to Agriculture			Variable:	Seasonality	
		Possible	Executed	Weight		
Range	Transitions	Transitions	Coefficient	Contrast	Significant?	
0	1000	11394	1784	0.85844755	0.92880423	yes
1000	2000	70659	6353	0.22766794	0.35490927	yes
2000	4000	86141	6527	0.0411512	0.06887971	yes
4000	5000	20136	737	-0.72799516	-0.78150202	yes
5000	6000	22338	468	-1.30200908	-1.38674832	yes
6000	7000	7112	17	-3.4915385	-3.52623911	yes
7000	8000	18	0	0	0	~
		217798	15886			
Transition:	Natural Grasslands to Agriculture			Variable:	Index_marginalization	
		Possible	Executed	Weight		
Range	Transitions	Transitions	Coefficient	Contrast	Significant?	
	1	42734	861	-0.89935616	-1.19310016	yes
	2	41320	2333	0.16887258	0.26851118	yes

	3	25393	1647	0.3164872	0.41883826	yes
	4	9765	836	0.61651477	0.69125957	yes
	5	1198	116	0.75196957	0.76271197	yes
		120410	5793			
Transition:	Natural Grasslands to Agriculture			Variable:	Potected_Areas_distance	
		Possible	Executed	Weight		
Range	Transitions	Transitions	Coefficient	Contrast	Significant?	
0	1000	650	17	-0.63231123	-0.63491039	yes
1000	2000	243	9	-0.27315069	-0.27363956	no
2000	3000	277	15	0.12465154	0.12495574	no
3000	7000	1244	82	0.33376715	0.33783345	yes
7000	8000	359	35	0.75955039	0.76277968	yes
8000	10000	697	91	1.08892536	1.09945746	yes
10000	20000	3681	365	0.77832868	0.81405034	yes
20000	177000	51101	4135	0.55500924	1.27881347	yes
177000	178000	278	4	-1.2418879	-1.24359061	yes
178000	179000	307	1	-2.73863926	-2.74113995	yes
179000	180000	314	3	-1.65623478	-1.65843385	yes
180000	181000	308	5	-1.11934905	-1.12113265	yes
181000	182000	329	4	-1.41258498	-1.41473381	yes
182000	183000	368	3	-1.81633922	-1.81901082	yes
183000	184000	360	6	-1.0925916	-1.09464866	yes
184000	185000	332	1	-2.81717253	-2.81989195	yes
185000	186000	350	7	-0.90687445	-0.90866243	yes
186000	188000	709	9	-1.36890991	-1.37348113	yes
188000	192000	1388	27	-0.93519229	-0.94246599	yes
192000	193000	324	9	-0.57040222	-0.57159948	no
193000	194000	327	13	-0.19949779	-0.19999449	no
194000	195000	303	17	0.16216738	0.16260788	no
195000	202000	2029	85	-0.14490589	-0.14723061	no
202000	203000	271	1	-2.61347612	-2.61566193	yes
203000	208000	1508	12	-1.84069767	-1.85176214	yes
208000	209000	326	5	-1.17705737	-1.17899844	yes
209000	210000	318	3	-1.66901451	-1.67124857	yes
210000	213000	973	13	-1.31703808	-1.32320247	yes
213000	215000	593	5	-1.78234319	-1.78662304	yes
215000	219000	1118	14	-1.38269205	-1.38995119	yes
219000	222000	937	16	-1.06792547	-1.07322761	yes
222000	223000	320	1	-2.78024526	-2.78285969	yes
223000	224000	325	3	-1.69099341	-1.69328872	yes

224000	233000	3014	43	-1.25050792	-1.26932046	yes
233000	234000	293	15	0.06537493	0.06553921	no
234000	235000	298	11	-0.2766411	-0.27724758	no
235000	236000	312	7	-0.78945578	-0.79091128	yes
236000	237000	318	10	-0.44256885	-0.44353196	no
237000	240000	1076	24	-0.79544872	-0.80051796	yes
240000	241000	382	3	-1.85397807	-1.85677222	yes
241000	242000	428	7	-1.11177684	-1.11424762	yes
242000	244000	858	18	-0.85808429	-0.862328	yes
244000	245000	467	7	-1.2003705	-1.20318285	yes
245000	246000	442	9	-0.88856731	-0.89079745	yes
246000	248000	948	27	-0.54467733	-0.54807355	yes
248000	249000	438	16	-0.28747075	-0.2883936	no
249000	250000	421	11	-0.63331604	-0.63499894	yes
250000	265000	6608	141	-0.84076187	-0.87419802	yes
265000	266000	423	3	-1.95669658	-1.95984969	yes
266000	268000	815	11	-1.30675815	-1.31189689	yes
268000	269000	449	3	-2.01676082	-2.02014163	yes
269000	270000	437	8	-0.99706953	-0.99943753	yes
270000	271000	440	3	-1.99637506	-1.99967705	yes
271000	272000	453	6	-1.32585328	-1.32872458	yes
272000	283000	4490	79	-1.037463	-1.06297681	yes
283000	284000	388	11	-0.54940407	-0.55079806	no
284000	285000	404	6	-1.20974669	-1.2121889	yes
285000	289000	1554	36	-0.75668418	-0.76378298	yes
289000	290000	362	12	-0.38808066	-0.38906537	no
290000	292000	750	13	-1.05269269	-1.05689696	yes
292000	293000	407	9	-0.80428158	-0.80620525	yes
293000	295000	775	12	-1.16740554	-1.17201113	yes
295000	296000	372	9	-0.71223241	-0.7138497	yes
296000	298000	772	28	-0.29489068	-0.29655789	no
298000	300000	729	19	-0.63588015	-0.63880875	yes
300000	307000	2457	52	-0.84911562	-0.86130494	yes
307000	308000	341	4	-1.44884273	-1.45109656	yes
308000	311000	979	7	-1.94849981	-1.95580731	yes
311000	312000	341	1	-2.84399977	-2.84679795	yes
312000	313000	301	4	-1.32249193	-1.32439581	yes
313000	314000	293	2	-1.99523024	-1.99742706	yes
314000	315000	285	0	0	0	~
315000	375000	9660	40	-2.49777425	-2.57850977	yes
375000	376000	110	4	-0.29219889	-0.29243341	no

376000	377000	100	2	-0.90687445	-0.90738454	no
377000	378000	102	1	-1.63017467	-1.63088362	no
378000	379000	93	3	-0.41625154	-0.41651907	no
379000	383000	320	7	-0.8153472	-0.81687268	yes
383000	384000	71	2	-0.55601348	-0.55627036	no
384000	394000	843	18	-0.84006579	-0.84417766	yes
394000	395000	74	0	0	0	~
395000	401000	395	2	-2.29571659	-2.29880599	yes
401000	402000	51	0	0	0	~
402000	403000	39	2	0.06717511	0.06719755	no
403000	404000	30	2	0.34588851	0.3459895	no
404000	405000	18	1	0.1517325	0.15175681	no
405000	406000	15	3	1.59865148	1.59906478	yes
406000	407000	14	0	0	0	~
407000	408000	15	1	0.34588851	0.345939	no
408000	409000	13	0	0	0	~
409000	410000	11	1	0.68236075	0.68244614	no
410000	411000	6	0	0	0	~
411000	520000	129	1	-1.86708442	-1.86802917	no
520000	521000	4	0	0	0	~
521000	621000	10	0	0	0	~
		120410	5793			
Transition:	Natural Grasslands to Agriculture			Variable:	distance_cities	
		Possible	Executed	Weight		
Range	Transitions	Transitions	Coefficient	Contrast	Significant?	
0	2000	815	143	1.4416517	1.46088823	yes
2000	5000	4844	635	1.09771004	1.17698318	yes
5000	8000	8708	887	0.81234269	0.90870255	yes
8000	9000	3470	275	0.53649414	0.55708259	yes
9000	16000	24967	1606	0.31174406	0.41018387	yes
16000	17000	3333	149	-0.07288183	-0.07489007	no
17000	25000	23939	902	-0.25117697	-0.30560468	yes
25000	47000	31324	977	-0.44690088	-0.56906728	yes
47000	48000	858	6	-1.96676165	-1.97318812	yes
48000	49000	935	15	-1.12725806	-1.13271871	yes
49000	50000	890	9	-1.59476764	-1.60092813	yes
50000	51000	874	4	-2.39313344	-2.40006557	yes
51000	52000	801	7	-1.7421079	-1.74785053	yes
52000	53000	757	1	-3.63897597	-3.64542616	yes
53000	54000	752	4	-2.24204321	-2.24790238	yes

54000	58000	2508	25	-1.60928155	-1.62685632	yes
58000	77000	6615	97	-1.21854647	-1.26017757	yes
77000	79000	372	3	-1.82311895	-1.82582586	yes
79000	80000	171	0	0	0	~
80000	96000	1412	7	-2.31281702	-2.32394698	yes
96000	97000	64	2	-0.4449218	-0.44511632	no
97000	98000	66	1	-1.18532186	-1.18571613	no
98000	99000	53	0	0	0	~
99000	119000	782	7	-1.71788747	-1.72346304	yes
119000	120000	37	31	0.12686453	0.12690588	no
120000	121000	168	0	0	0	~
121000	144000	895	0	0	0	~
		120410	5793			
Transition:	Natural Grasslands to Agriculture			Variable:	distance_roads	
		Possible	Executed	Weight		
Range	Transitions	Transitions	Coefficient	Contrast	Significant?	
0	1000	6721	804	0.98896027	1.08537073	yes
1000	2000	13065	1227	0.71820331	0.84720097	yes
2000	3000	12196	924	0.48358086	0.55381935	yes
3000	5000	16010	985	0.26011672	0.30597152	yes
5000	6000	9234	414	-0.07396533	-0.07989149	no
6000	7000	5900	218	-0.27561766	-0.28810444	yes
7000	8000	6426	162	-0.67003206	-0.69787053	yes
8000	86000	50858	1059	-0.86572399	-1.23385701	yes
		120410	5793			
Transition:	Natural Grasslands to Agriculture			Variable:	altitude	
		Possible	Executed	Weight		
Range	Transitions	Transitions	Coefficient	Contrast	Significant?	
0	100	2316	370	1.32489141	1.37376802	yes
100	200	207	49	1.81414493	1.8212599	yes
200	300	691	30	-0.10763679	-0.10822855	no
300	400	693	13	-0.97222377	-0.97592779	yes
400	500	946	25	-0.62166454	-0.62540778	yes
500	600	518	24	-0.03956202	-0.03972992	no
600	1000	1972	53	-0.60434791	-0.61204174	yes
1000	1100	2826	45	-1.1389837	-1.15574865	yes
1100	1200	5379	691	1.07029816	1.15555328	yes
1200	1300	8067	165	-0.88400603	-0.92654622	yes
1300	1900	61568	1880	-0.47293955	-0.81617674	yes

1900	2000	10335	701	0.36437377	0.40555327	yes
2000	2200	15631	1294	0.57981426	0.69897579	yes
2200	2300	4509	290	0.30744718	0.32129896	yes
2300	2400	2899	123	-0.1316623	-0.13471969	no
2400	2500	1147	27	-0.74032743	-0.74547573	yes
2500	2600	304	4	-1.33256844	-1.33449863	yes
2600	3700	122	1	-1.81087088	-1.81175451	no
3700	3800	13	0	0	0	~
3800	4900	109	0	0	0	~
		120407	5793			
Transition:	Natural Grasslands to Agriculture			Variable:	Potential_evapotranspiration	
		Possible	Executed	Weight		
Range	Transitions	Transitions	Coefficient	Contrast	Significant?	
0	1000	394	61	1.28767722	1.29535344	yes
1000	4000	25812	2779	0.87010902	1.29915166	yes
4000	5000	35360	1542	-0.10296709	-0.14310259	yes
5000	6000	46064	1200	-0.63636829	-0.90088711	yes
6000	7000	12339	192	-1.16239629	-1.24071721	yes
7000	8000	441	19	-0.11562049	-0.1160239	no
		120410	5793			
Transition:	Natural Grasslands to Agriculture			Variable:	GDP	
		Possible	Executed	Weight		
Range	Transitions	Transitions	Coefficient	Contrast	Significant?	
0	100	9744	262	-0.62373029	-0.6672337	yes
100	200	10135	350	-0.36559698	-0.39551559	yes
200	400	15006	652	-0.12666362	-0.14465048	yes
400	500	13661	466	-0.37833187	-0.42104362	yes
500	700	8236	402	-0.00470068	-0.00506577	no
700	800	8659	294	-0.20570532	-0.2095337	no
800	900	3558	27	-1.90842378	-1.93668537	yes
900	1000	2841	166	0.18535888	0.1905227	yes
1000	1100	5549	593	0.8419159	0.90725799	yes
1100	1200	1986	135	0.34686921	0.35409539	yes
1200	1300	3673	97	-0.64221341	-0.65824333	yes
1300	1400	3866	271	0.3798953	0.39586747	yes
1400	1500	1188	305	1.90206232	1.94998289	yes
1500	1600	3976	169	-0.14962229	-0.15466557	no
1600	1700	1280	137	0.84364501	0.85785795	yes
1700	2000	2713	211	0.49208819	0.50721584	yes

2000	2100	1916	39	-0.90879264	-0.91925272	yes
2100	2200	47	17	2.39709171	2.39986027	yes
2200	2300	677	136	1.58431136	1.60393566	yes
2300	2400	564	71	1.02724645	1.03547026	yes
2400	2500	43	9	1.6359398	1.63723725	yes
2500	2600	93	5	0.09717685	0.09725973	no
2600	2700	1231	118	0.72094602	0.73196088	yes
2700	2800	4342	27	-2.10893999	-2.1447025	yes
2800	2900	113	28	1.854629	1.85886551	yes
2900	3000	1348	3	-3.14046125	-3.1524048	yes
3000	3200	9	1	0.88563421	0.88573931	no
3200	3300	360	88	1.8366105	1.8499639	yes
3300	3600	1061	116	0.86748101	0.87969453	yes
3600	3700	529	37	0.37751494	0.37960749	yes
3700	3800	1048	2	-3.29450572	-3.30384028	yes
3800	4700	1588	22	-1.30016167	-1.31076493	yes
4700	4800	1	0	0	0	~
4800	4900	13	1	0.4801691	0.48023731	no
4900	5000	307	4	-1.3623627	-1.36444498	yes
5000	5100	115	6	0.06548734	0.06555543	no
5100	5200	771	4	-2.29111669	-2.29749891	yes
5200	5400	24	2	0.56718048	0.56733537	no
5400	5500	412	61	1.21516339	1.22289179	yes
5500	5600	4	0	0	0	~
5600	5800	1	1	0	0	~
5800	6000	12	1	0.56718048	0.56725791	no
6000	6400	306	35	0.91830499	0.92208261	yes
6400	6500	78	6	0.4801691	0.48057867	no
6500	6600	187	97	3.03997706	3.05664837	yes
6600	6700	406	43	0.83187303	0.83624072	yes
6700	6900	38	1	-0.64584216	-0.64600454	no
6900	7400	7	7	0	0	~
7400	7600	1009	68	0.33764031	0.34116213	yes
7600	7800	1490	20	-1.33220966	-1.34227416	yes
7800	8100	1	0	0	0	~
8100	8400	1424	57	-0.21224682	-0.21468525	no
8400	9000	70	1	-1.26903076	-1.26948836	no
9000	9400	165	4	-0.73003426	-0.7308043	no
9400	9600	162	27	1.35563784	1.35923307	yes
9600	9700	39	1	-0.67251041	-0.67268201	no
9700	9800	146	2	-1.31159037	-1.31256135	no

9800	10100	2	0	0	0	~
10100	10600	36	5	1.14052646	1.14113525	yes
10600	11800	336	35	0.81331355	0.8168138	yes
11800	11900	25	4	1.30684767	1.3073697	yes
11900	12400	35	1	-0.56128478	-0.56141948	no
12400	12800	10	6	3.37054086	3.3715777	yes
12800	13900	643	0	0	0	~
13900	15300	73	7	0.72133116	0.72197516	no
15300	16300	733	2	-2.93619053	-2.94259638	yes
16300	20800	56	1	-1.04225744	-1.04258586	no
20800	21300	28	3	0.84481221	0.84511837	no
21300	21900	106	2	-0.98616797	-0.98676967	no
21900	26800	7	0	0	0	~
26800	28500	23	1	-0.1259667	-0.12599072	no
28500	29100	69	21	2.13839718	2.14171756	yes
		120410	5793			
Transition:	Natural Grasslands to Agriculture			Variable:	Slope	
		Possible	Executed	Weight		
Range	Transitions	Transitions	Coefficient	Contrast	Significant?	
0	2	43363	3437	0.53254141	1.0041625	yes
2	4	22007	1306	0.22175717	0.27804532	yes
4	6	14964	499	-0.38191097	-0.42674704	yes
6	8	10725	216	-0.89973875	-0.95791506	yes
8	10	8143	124	-1.18431729	-1.2352185	yes
10	22	20091	206	-1.58487464	-1.73923587	yes
22	24	606	3	-2.31833476	-2.32309234	yes
24	36	511	2	-3.21350857	-3.21763834	yes
		120410	5793			
Transition:	Natural Grasslands to Agriculture			Variable:	Soils	
		Possible	Executed	Weight		
Range	Transitions	Transitions	Coefficient	Contrast	Significant?	
	Solonchak	2612	44	-1.08174719	-1.09678268	yes
	Vertisol	2585	209	0.55410641	0.56990355	yes
	Litosol	17172	161	-1.67526526	-1.80773676	yes
	Regosol	22574	445	-0.92162412	-1.05621372	yes
	Fluvisol	59	2	-0.36495824	-0.36511037	no
	Tfeozem	21100	1330	0.28595915	0.35745719	yes
	Xerosol	26556	1547	0.20202765	0.26654905	yes
	Yermosol	3796	255	0.35404494	0.36768032	yes

	Planosol	3270	544	1.37330448	1.44784624	yes
	Cambisol	4764	410	0.62225276	0.6569295	yes
	Rendzina	7397	132	-1.02307581	-1.06550905	yes
	Castagnozem	3767	294	0.51575156	0.53706611	yes
	Acrisol	326	68	1.4957941	1.50388197	yes
	Luvisol	2096	235	1.04758275	1.0746625	yes
	Gleysol	928	61	0.33078073	0.33377344	yes
	Chernozem	459	26	0.17230465	0.17301798	no
	Andosol	37	0	0	0	~
	Nitosol	4	0	0	0	~
	Solonetz	7	0	0	0	~
	Ranker	901	30	-0.38349875	-0.38593488	yes
		120410	5793			
Transition:	Natural Grasslands to Agriculture			Variable:	seasonality	
		Possible	Executed	Weight		
Range	Transitions	Transitions	Coefficient	Contrast	Significant?	
0	1000	649	134	1.63861874	1.65751849	yes
1000	2000	2422	288	0.98215289	1.01435238	yes
2000	5000	46786	3475	0.46213368	0.90346357	yes
5000	6000	19381	1203	0.26955221	0.32963703	yes
6000	7000	39192	498	-1.36789391	-1.68988306	yes
7000	8000	10809	195	-1.01198376	-1.07491925	yes
8000	9000	1171	0	0	0	~
		120410	5793			
